
JOURNAL INFORMATION
==============================
NLM Title Abbreviation: Insights Imaging
Journal ID: Insights Imaging
NLM Journal ID: 101532453

Insights into Imaging

EISSN: 1869-4101
Publisher: Springer

ARTICLE INFORMATION
==============================
PMCID: PMC8408290
PMID: 34467439
DOI: 10.1186/s13244-021-01061-y
Article ID: 1061
Article version: 1
Subjects: Meeting Abstracts

ESHNR 2021 Book of Abstracts

Electronic publication date: 2021 Sep 1
Publication date: 2021 Sep
Volume: 12
Issue: Suppl 4
Issue sponsor: Publication costs were not supported by sponsorship.
Electronic Location ID: 122
Copyright: © The Author(s) 2021
Copyright year: 2021
License: Open Access This article is licensed under a Creative Commons Attribution 4.0 International License, which permits use, sharing, adaptation, distribution and reproduction in any medium or format, as long as you give appropriate credit to the original author(s) and the source, provide a link to the Creative Commons licence, and indicate if changes were made. The images or other third party material in this article are included in the article’s Creative Commons licence, unless indicated otherwise in a credit line to the material. If material is not included in the article’s Creative Commons licence and your intended use is not permitted by statutory regulation or exceeds the permitted use, you will need to obtain permission directly from the copyright holder. To view a copy of this licence, visit http://creativecommons.org/licenses/by/4.0/.
License URL: https://creativecommons.org/licenses/by/4.0/

Conference name: ESHNR 2021 Annual Scientific Meeting
Conference location: Virtual
Conference date: 2-4 September 2021
issue-copyright-statement: © European Society of Radiology (ESR) 2021

==============================

eshnr
european society of
head and neck radiology

### Insights into Imaging

Education and strategies in European radiology

ESHNR 2021 Book of Abstracts / Volume 12 / Supplement 4 / September 2021

ESHNR 2021 / September 2–4 / Online Edition
33rd Annual Meeting & Refresher Course

ESR
European Society of Radiology eshnr
european society of
head and neck radiology

### ESHNR 2021
### Stay-at-home Edition
### 33rd Annual Meeting and Refresher Course

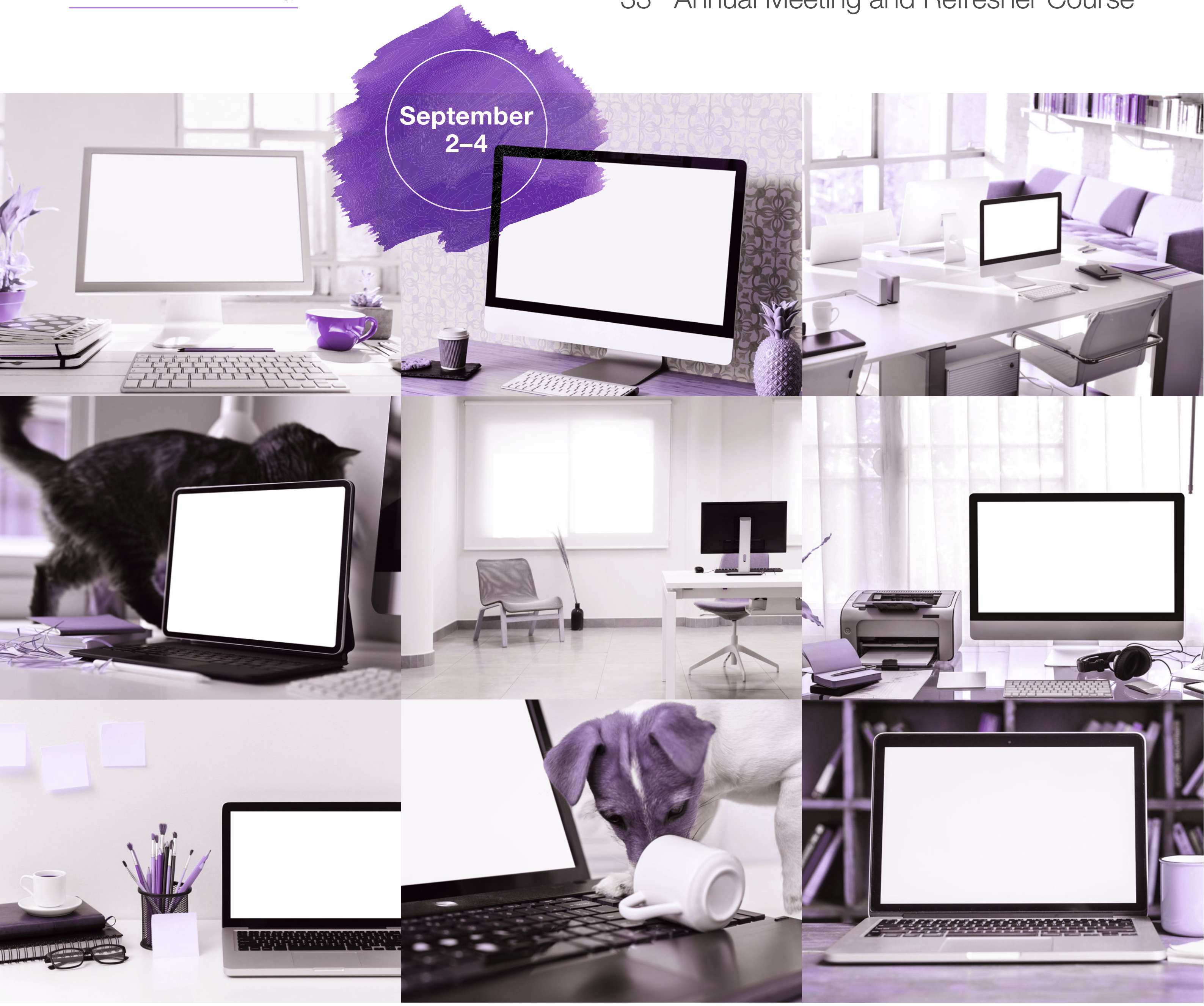

Insights Imaging (2021) 12 (Suppl 4):122
https://doi.org/10.1186/s13244-021-01061-y
Published: 1 September 2021

www.eshnr.eu

### Organising Secretariat

ESHNR Office
Am Gestade 1
1010 Vienna
office@eshnr.eu | www.eshnr.eu

### CME Credits

### Live Event

The ESHNR 2021 Annual Scientific Meeting, Vienna (ONLINE), Austria, 02/09/2021-04/09/2021 has been accredited by the European Accreditation Council for Continuing Medical Education (EACCME®) with 21 European CME credits (ECMEC®s). Each medical specialist should claim only those hours of credit that he/she actually spent in the educational activity.

### On-Demand Lectures

The On-Demand Lectures have been accredited by the European Accreditation Council for Continuing Medical Education (EACCME®) with 21 European CME credits (ECMEC®s). Each medical specialist should claim only those hours of credit that he/she actually spent in the educational activity.

Through an agreement between the Union Européenne des Médecins Spécialistes and the American Medical Association, physicians may convert EACCME® credits to an equivalent number of AMA PRA Category 1 Credits™. Information on the process to convert EACCME® credit to AMA credit can be found at www.ama-assn.org/education/earn-credit-participation-international-activities.

### Confirmation of Attendance

Each participant who successfully registered and attended the live event will receive a confirmation of attendance after the meeting. The CME credits are only available for those who successfully complete the evaluation.

### Disclaimer

The **ESHNR 2021 Book of Abstracts** summarises the scientific abstract presentations and posters accepted to be held at the Annual Scientific Meeting – Online Edition | September 2-4, 2021. Abstracts were submitted by the authors warranting that good scientific practice, copyrights and data privacy regulations have been observed and relevant conflicts of interest declared. Abstracts reflect the authors' opinions and knowledge. The ESHNR does not give any warranty about the accuracy or completeness of medical procedures, diagnostic procedures or treatments contained in the material included in this publication. The views and opinions presented in all given abstracts and presentations, including scientific, educational and professional matters, do not necessarily reflect the views and opinions of the ESHNR. In no event will the ESHNR be liable for any direct or indirect, special, incidental, consequential, punitive or exemplary damages arising from the use of these abstracts.

In preparing this publication, every effort has been made to provide the most current, accurate, and clearly expressed information possible. Nevertheless, inadvertent errors in information can occur. The ESHNR is not responsible for typographical errors, accuracy, completeness or timeliness of the information contained in this publication.

### Oral Presentations

### The concordance between the audiometry and ossicular findings in CT scan among the patients with chronic otitis media: A brief report

*Author: Mohammad Ali Kazemi*

**Keywords:** Chronic otitis media, ossicular fixation, audiometry
**Short summary:** Ossicular erosion in the temporal CT and type-B tympanogram seem to be associated with the audiometric findings of COM but tympanosclerosis, stapes sclerosis, and ossicular disruption seemed to make no difference in the audiometric findings.
**Purpose/Objectives:** The application of imaging modalities in comprehensive evaluation of the chronic otitis media (COM) is trending. A plausible association between the temporal CT scan abnormalities and impaired audiometry has been suggested. In this study, we attempted to identify the pathological features in the temporal CT scan of these patients and investigate their concordance with audiometric findings.
**Methods and materials:** This cross-sectional study evaluated 200 ears suffering from COM, candidates for both audiometry and temporal CT scan. The CT images were evaluated in coronal and axial slices.
**Result:** Tympanic cavity opacification more than 50 %, ossicular erosion, tympanosclerosis, and stapes sclerosis were present in 26 %, 21 %, 49 %, and 31 % of the subjects, respectively. Unlike ossicular erosion and type-B tympanogram, tympanosclerosis, stapes sclerosis, and ossicular disruption seemed to make no difference in the audiometric findings.
**Conclusion:** Ossicular erosion in the temporal CT and type-B tympanogram seem to be associated with the audiometric findings of COM.

### Comparison of vestibular hydrops grade and degree of hearing loss in PTA of patients with Ménière's disease

*Author: Mohammad Ali Kazemi*

**Keywords:** Ménière's disease, Pure tone audiometry, MRI
**Short summary:** MRI has acceptable diagnostic value in evaluation of Ménière's disease. Significant correlation between grade of vestibular hydrops and degree of hearing loss was found.
**Purpose/Objectives:** To evaluate concordance of grading of vestibular hydrops in MRI and degree of hearing loss in PTA of patients with Ménière's disease.
**Methods and materials:** In a cross-sectional prospective study 19 patients with mean age of 50.6 +-12.1years (24 ears) with definite Ménière's disease underwent 3T-MRI for hydrops imaging. Their correlation with PTA functional test and control group (29 ears) was evaluated.
**Result:** Sesitivity, specifity, positive predictive value and negative predictive value for MRI findings were as follows: vestibular hydrops (100 %, 92.3 %, 96 % and 100 % respectively), for choclear hydrops (79.2 %, 92.3 %, 95 % and 70.6 % respectively), absence of vestibular aquiduct visiblity ( 91.7 %, 69.2 %, 84.6 % and 81.8 % respectively) and choclear enhancement (58.3 %, 76.9 % 82.4 % and 50 % respectively). Hearing loss range in patients with was 14.3+/-5.35 in vestibular hydrops grade 1, 33.08 +/- 24.62 in grade 2 and 47.5+/- 9.57 in grade 3.
**Conclusion:** Significant correlation between grade of vestibular hydrops in MRI and degree of hearing loss was found.

### Measurement of carotid artery calcifications in cone-beam computed tomography using different software programs

*Author: Nagihan Koc*

**Keywords:** cone beam computed tomography, carotid atherosclerosis, calcifications, software
**Short summary:** Atherosclerotic plaque in the carotid artery is an important risk factor for a cerebrovascular accident or ischemic stroke. One of the most common locations for calcified carotid plaques is the cervical portion of the artery, which is also considered as the extracranial segment of the internal carotid artery. Based on their anatomic location, calcifications in this region can often be detected on routine maxillofacial imaging. The presence and course of calcifications can be identified in cone-beam computed tomography (CBCT) scans. Furthermore, calcification volume and area also can be quantified on CBCT images by using software packages.
**Purpose/Objectives:** The aim of this study was to compare the volume and area measurements of carotid artery calcifications (CAC) performed with different CBCT software.
**Methods and materials:** CBCT images of 44 patients with CAC were included in a retrospective study. A total of 64 calcifications were recorded (24 unilateral and 20 bilateral). The calcification volume ($mm^3$) and surface area ($mm^2$) were calculated separately with the 3D-DOCTOR software and the Mimics Medical software. The intraclass correlation coefficient was applied to assess intraobserver agreement. The difference between measurements were evaluated with the t-test. All analyses were performed using VassarStat Computation Website (http://vassarstats.net/) and p-values < 0.05 were considered statistically significant.
**Result:** Intraobserver agreement was excellent between two software packages in volume and surface area measurements ($\geq 0.90$). Significant differences were observed in the measurements of calcification volume ($52.97 \pm 48.97$ $mm^3$ vs. $39.70 \pm 34.40$ $mm^3$) and surface area ($109.55 \pm 85.40$ $mm^2$ vs. $98.99 \pm 75.34$ $mm^2$) between software programs. The measurements obtained with Mimics software were significantly higher than in those obtained with 3D-DOCTOR software ($p < 0.05$).
**Conclusion:** Different software packages may influence the quantification of CAC in CBCT images.

### The Relationship Between Koos Classification and ADC-Signal Intensity Values of Vestibular Schwannoma

*Authors: Umut Percem Orhan Soylemez, Basak Atalay, Nesrin Gunduz*

**Keywords:** vestibular schwannoma, Koos classification, Magnetic Resonance, Apparent diffusion coefficient, Signal intensity
**Short summary:** Vestibular schwannomas which are the most common cerebellopontine angle (CPA) tumors, can be detected with a great accuracy in MRI. Surgery and radiotherapy are treatment options also observation may be choosen. Koos classification, which deals with extension to the internal acoustic canal and compression to brainstem is used to guide the treatment of these non-malignant tumors. In this classification, schwannomas are classified in 4 grades as: small intracanalicular tumor; grade 1, small tumor with protrusion into the cerebellopontine angle but no contact with the brainstem; grade 2, tumor occupying the cerebellopontine cistern with no brainstem displacement; grade 3 and large tumor with brainstem and cranial nerve displacement; grade 4.
**Purpose/Objectives:** Reliability of Apparent diffusion coefficient (ADC) and signal intensity (SI) according to tumor grade have not been well studied. This study evaluates ADC and SI measurements between vestibular schwannoma grades according to Koos classification and also the interobserver reliability of Koos classification.
**Methods and materials:** The images of patients who underwent contrast enhanced temporal MRI with the diagnosis of vestibular schwannoma between 2017 and 2021 were retrospectively scanned. The keywords; 'vestibular schwannoma', 'acoustic neuroma' and 'cerebellopontine angle tumor' were entered into the system and scanned. Images with inadequate quality for assesment and tumors which have incompatible features with schwannoma were excluded from the study. The side, maximum diameter of tumor, ADC and SI values (in contrast enhanced images), Koos class of tumor were assessed by two radiologists blinded to the clinical and pathological data. Cohen's Kappa was used to analyse interobserver agreement of Koos grade, Kendall's tau b correlation coefficient was used to analyse correlation between ADC, SI measurements and Koos grade of tumors.
**Result:** 33 patients [21(61.8 %) females, 52.6±16.6 years] with 34 schwannomas were included. Seventeen of the tumors were right sided. The mean of the maximum diameter was 15.03±7.13 mm. The means of ADC and SI values were 1402±363 and 1400±512, respectively. The frequencies for each tumor grade from 1 to 4 were, 3 (8.8 %), 13 (38.2 %), 9 (26.5 %), and 9 (26.5), respectively. The reliability analyses revealed excellent inter-observer agreement for ADC (ICC: 0.93, 95 % CI: 0.87-0.97, p<0.001) and SI (ICC: 0.98, 95 % CI: 0.96-0.99, p<0.001). The percentage agreement for tumor grading was 97 % (k= 0.96, p<0.001). The maximum size (p<0.001) and ADC (p=0.039) differed significantly between the tumor grades. A correlation was not found between the Koos grade and ADC ($\tau_b$: 0.18).
**Conclusion:** MR is an excellent diagnostic method for schwannomas. In addition to recognizing these tumors, it is very important to indicate the part in the acoustic canal, whether they are compressing the brain stem or not, that is, classifying them. Koos classification is a practical and useful grading system with excellent interobserver aggreement for vestibular schwannomas. ADC values differs significantly between the tumor grades, ADC is a measurement method that can be used not only in the differential diagnosis of CPA tumors but also in the grading schwannomas.

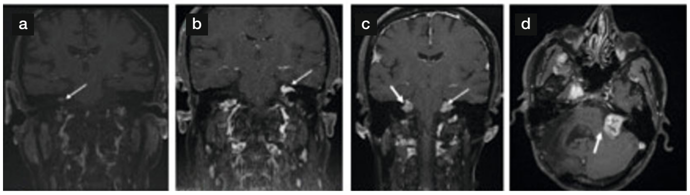

**a.** Right sided small intracanalicular tumor (arrow), grade 1; **b.** Left sided tumor which extends to cerebellar cistern but doesn't touch the brainstem (arrow), grade 2; **c.** In an NF2 patient bilateral tumors that touch the brainstem but there is no compression (arrow), grade 3; **d.** In a different NF2 patient (right side was operated), left sided tumor that compresses the brainstem and cerebellum (arrow), grade 4

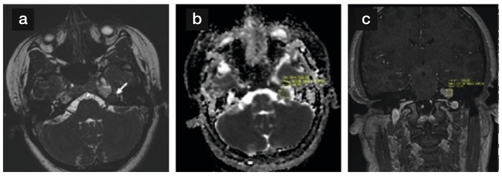

**a.** Left sided Koos grade 2 schwannoma in axial FIESTA sequence (arrow);
**b.** ADC measurement with region of interes;
**c.** Signal intensity measurement in coronal contrast enhanced image

### The role of conventional, diffusion-weighted and dynamic contrast-enhanced MR imaging in the evaluation of parotid tumors and its value in predicting histopathological type

*Authors: Nick Janssen, Anja Bernaerts, Joost van Dinther, Filip Deckers, Bert De Foer*

**Keywords:** parotid tumours, MRI, DWI, DCE-MRI
**Short summary:** The role of conventional, diffusion-weighted and dynamic contrast-enhanced MR imaging in the evaluation of parotid tumors and its value in predicting histopathological type.
**Purpose/Objectives:** This retrospective study provides a summary using the current knowledge of the role of conventional MRI, diffusion weighted imaging (DWI) and dynamic contrast enhanced MRI (DCE-MRI) in the diagnosis and histopathological characterization of parotid tumors.
**Methods and materials:** Ethical approval was obtained. Patients from July 2019 to April 2021 with a parotid tumor who were evaluated with conventional MRI, DWI and DCE-MRI and confirmed by histopathology were included in this study. Different morphologic features, signal intensities, perineural spread, presence of lymphadenopathies, apparent diffusion coefficient (ADC) and time-intensity curves (TIC) were taken into account. Wash-out rates were determined at 300 seconds after gadolinium-contrast administration. We classified the various histopathological types as follows: pleomorphic adenomas, Warthin tumors, other benign tumors and malignant tumors. Multiple comparisons were made to determine if any differences between the various histopathological types existed. In total 27 patients were included, consisting of 10 pleomorphic adenomas, 8 Warthin tumors, 1 myoepithelioma and 8 malignant tumors.
**Result:** Benign tumors showed well-defined and smooth margins, some malignant tumors showed irregular margins, local invasiveness, perineural spread and/or presence of lymphadenopathies. Great overlap was seen in ADC values between Warthin tumors and malignant tumors. Little overlap was seen in ADC values between pleomorphic adenomas and Warthin tumors or malignant tumors. DCE-MRI shows that TIC patterns are often very specific for a histopathological type. All Warthin tumors showed a fast peak with wash-out rate of 50 % or more. All pleomorphic adenomas demonstrated a gradual peak with plateau. The malignant tumors also had a fast peak but high variability in wash-out rate, ranging from plateau up to nearly 40 % wash-out rate. No differentiation could be made between myoepithelioma and pleomorphic adenomas.
**Conclusion:** The combination of conventional MRI, DWI and DCE-MRI can contribute in determining the histopathological type of parotid tumors and the discrimination of malignancies.

### Measuring 3D cochlear duct length on MRI: Is it accurate and reliable?

*Authors:* *Mehmet Bilgin Eser, Basak Atalay, Mahmut Bilal Dogan, Nesrin Gunduz, Mahmut Tayyar Kalcioglu*

**Keywords:** Cochlear duct, Organ of Corti, Hearing loss, Magnetic resonance imaging, Computed tomography

**Short summary:** The cochlear implant is an effective treatment for patients with profound hearing loss. Therefore, the number of patients undergoing pre-surgical imaging is increasing. In the last 25 years, many studies have been conducted on cochlea length measurements using computed tomography (CT) and cone-beam CT. The studies' ultimate aim was to select the most suitable cochlear implants for the patient-specific anatomy and tonotopy. The radiation exposure during these CT scans is relatively high, so making measurements by MRI may be an alternative.

**Purpose/Objectives:** This study aimed to test the repeatability and reliability of cochlea lateral wall length (LWL) measurements using 3D magnetic resonance imaging.

**Methods and materials:** Two observers measured the cochlea LWL of patients with post-lingual hearing loss using computed tomography (CT) and magnetic resonance imaging (MRI). Intra-class correlation coefficient (ICC) was used to test the intra-observer and interobserver agreement of the 3D cochlea measurements.

**Result:** Among the 35 patients included, 21 (70 %) were male. The mean age of the participants was 39.85 ± 16.60 years. Observer 1 measured the mean LWL as 41.52 ± 2.25 mm on CT and 41.44 ± 2.18 mm on MR, and the mean difference was found to be 0.08 (95 % CI: -0.11 - 0.27) mm. Observer 2 measured the mean LWL as 41.74 ± 2.69 mm on CT and 42.33 ± 2.53 mm on MR, and the mean difference was found to be -0.59 (95 % CI: -1.00 and -0.20) mm. In the inter-observer agreement for LWL was 0.90 (95 % CI: 0.84 - 0.94) for CT, while ICC was 0.69 (95 % CI: 0.46 - 0.82) in MRI was found.

**Conclusion:** This study showed that the 3D measurements of cochlea made by CT showed "excellent" intra-observer and interobserver agreement. Intra-observer agreement for LWL measurements was "good" to "excellent," while the interobserver agreement was "moderate" for MRI. These results confirmed that the 3D cochlea measurement method using CT imaging is the reference method, and MRI may be an alternative with similar reliable results with CT.

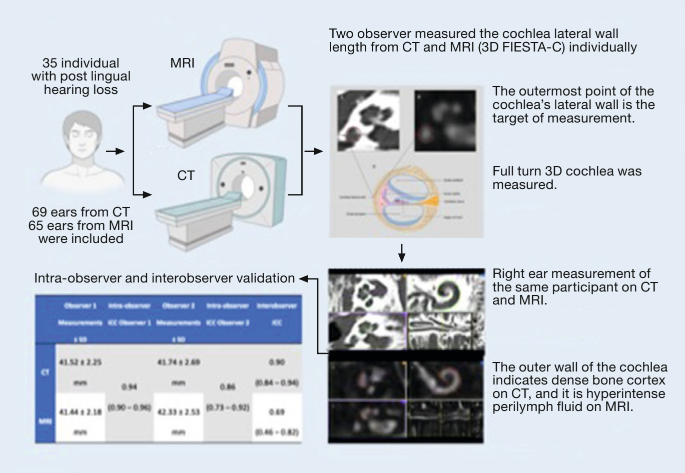

### MRI findings in acute tonsillar infections

*Authors:* *Jaakko Heikkinen, Janne Nurminen, Jarno Velhonoja, Heikki Irjala, Tero Soukka, Tatu Happonen, Mikko Nyman, Kimmo Mattila, Jussi Hirvonen*

**Keywords:** Magnetic resonance imaging, Peritonsillar abscess, Emergency medicine, Infection

**Short summary:** This paper aims to describe MRI findings regarding tonsillar infections and to investigate the significance of imaging findings related to clinical treatments and outcomes. MRI provides superior soft-tissue contrast to CT, and emergency neck MRI is feasible even in acutely ill patients. We found high diagnostic accuracy in detecting abscesses and parapharyngeal and retropharyngeal deep extension, with positive predictive value for abscesses (0.98) and deep extension (0.87). We found no evidence of intratonsillar abscesses. Patients with large abscesses and widespread edema patterns had a more severe course of illness. These results suggest that MRI may provide additional value in the management of these patients.

**Purpose/Objectives:** Previous literature is vague on the prevalence and exact nature of abscesses in tonsillar infections ranging from intratonsillar and peritonsillar collections to deep extension involving parapharyngeal and retropharyngeal spaces. Magnetic resonance imaging (MRI) has excellent diagnostic accuracy in detecting neck infections and can potentially clarify this issue. We sought to characterize the spectrum of MRI findings regarding tonsillar infections.

**Methods and materials:** A retrospective cohort study of emergency neck MRI scans of patients with tonsillar infections. Imaging data were assessed in terms of signs of infection and the location of abscesses and were compared with clinical findings, final diagnosis, and surgical findings as reference standards.

**Result:** The study included 132 patients with tonsillar infection. Of these, 110 patients (84 %) had one or more abscesses (99 unilateral, 11 bilateral, average volume 3.2 mL). Most abscesses were peritonsillar, and we found no evidence of intratonsillar abscesses. Imaging showed evidence of parapharyngeal and retropharyngeal extension in 36 % and 12 % of patients, respectively. MRI had a high positive predictive value for both abscesses (0.98) and deep extension (0.87). Patients with large abscesses and widespread edema patterns had a more severe course of illness.

**Conclusion:** Emergency neck MRI can accurately describe the extent and nature of abscess formation in tonsillar infections. These results suggest that MRI may provide additional value in the management of these patients.

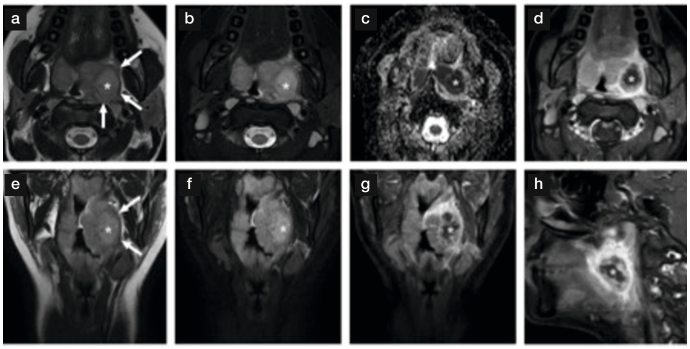

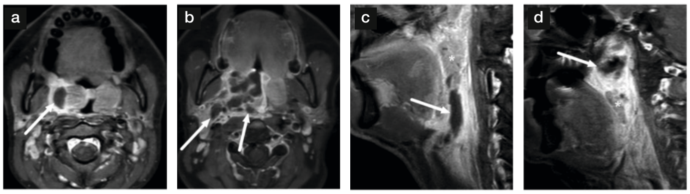

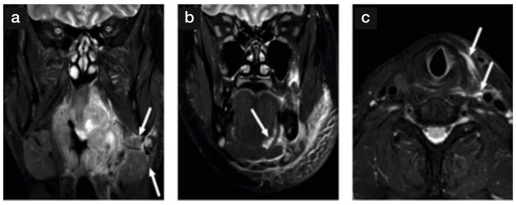

### PET/MR vs. PET/CT for locoregional staging of oropharyngeal squamous cell cancer

*Authors: Secil Telli Erdogan, Karin Söderkvist, Lennart Flygare*

**Keywords:** Head and neck cancer, PET-MR, PET-CT, 18F-FDG, Cancer staging
**Short summary:** The value of fluorine-18-fluorodeoxyglucose positron emission tomography/computed tomography (18F-FDG PET/CT) for TN-staging in Head & neck cancer (HNC) has been proven in numerous studies. A few studies have investigated the value of 18F-FDG PET/MRI in staging of HNC and the combined results indicate a potential for PET/MRI but the scientific evidence remains weak. We compared PET/CT and PET/MRI to a reference standard for TN-staging of oropharyngeal carcinomas and found no significant differences with moderate to substantial agreement between methods.
**Purpose/Objectives:** To compare performance of PET/CT and PET/MRI for locoregional staging in patients with oropharyngeal carcinomas.
**Methods and materials:** Primary pre-therapeutic 18F-FDG PET/CT and 18F-FDG PET/MRI examinations from 40 individuals with oropharyngeal carcinomas were retrospectively reviewed by two radiologists independently of each other. TN-stage and primary tumour size was noted. The results were compared between observers, modalities and against TN-stage previously set at a multidisciplinary conference (MDC). Linear Weighted Kappa ($\kappa$) was used to assess interrater reliability. Wilcoxon matched-pair signed-rank test was used to test for systemic differences between observers or methods in staging and tumour size measurements. A P-value below 0.05 considered statistically significant.
**Result:** Moderate to substantial agreement ($\kappa$ = 0.50-0.63) was found between PET/CT and PET/MRI for T- and N-stage for both observers. Substantial interobserver-agreement for nodal staging with both PET/CT ($\kappa$ = 0.75) and PET/MRI ($\kappa$ = 0.80) and for tumour staging with PET/MRI ($\kappa$ = 0.67). Moderate agreement for tumour staging with PET/CT ($\kappa$ = 0.45). Lower agreement between PET/MRI and MDC regarding tumour staging (fair to moderate, $\kappa$ =0.33-0.48) than for PET/CT and MDC (moderate to substantial, $\kappa$ = 0.57-0.65). Comparable agreement between PET/MRI and MDC for nodal staging ($\kappa$ = 0.46-0.65) as compared to PET/CT and MDC ($\kappa$ = 0.44-0.60). No systematic significant differences in staging nor in measurement of maximum tumour diameter was found.
**Conclusion:** The results indicate that 18F-FDG PET/MRI may be a viable alternative to 18F-FDG PET/CT for TN-staging and assessment of maximal tumour diameter in oropharyngeal squamous cell cancer.

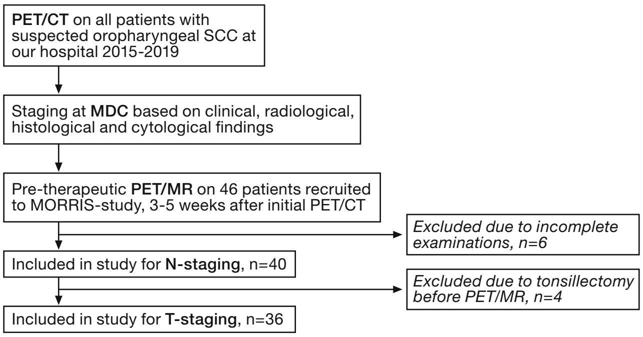

Interrater reliability between PET/MR and PET/CT or Observers 1, 2 and Multidisciplinary Conference for Tumour staging in 40 patients with oropharyngeal carcinoma.

| Evaluation | Concordance | Cohen´s Kappa | 95 % CI | Kappa with linear weighting |
|---|---|---|---|---|
| **PET/MR / PET/CT** | | | | |
| Obs 1 | 0.61 | 0.45 | 0.23–0.67 | 0.60 |
| Obs 2 | 0.61 | 0.40 | 0.17–0.64 | 0.50 |
| **PET/CT** | | | | |
| Obs 1/Obs 2 | 0.55 | 0.35 | 0.13–0.58 | 0.45 |
| Obs 1/MDC | 0.63 | 0.47 | 0.26–0.67 | 0.57 |
| Obs 2/MDC | 0.68 | 0.53 | 0.32–0.73 | 0.65 |
| **PET/MR** | | | | |
| Obs 1/Obs 2 | 0.70 | 0.57 | 0.37–0.77 | 0.67 |
| Obs 1/MDC | 0.50 | 0.28 | 0.06–0.50 | 0.48 |
| Obs 2/MDC | 0.50 | 0.25 | 0.02–0.48 | 0.33 |

CI = Confidence Interval, Obs = Observer, MDC = MultiDisciplinary Conference

### Diagnostic value of cone-beam CT-scan in the assessment of otosclerosis

*Authors: Valérie Gille, Anja Bernaerts, Jan Casselman, Joost van Dinther, Erwin Offeciers, Andrzej Zarowski, Bert De Foer*

**Keywords:** Otospongiosis, Cone beam Computed Tomography, Stapedotomy
**Short summary:** In patients with otosclerosis leading to substantial conductive hearing loss, management by means of stapedotomy is the preferred surgical intervention. Pre-operative imaging consists of a temporal bone CT-scan in order to confirm the diagnosis, to assess contra-indications and improve patient counseling. In this study we retrospectively evaluated the diagnostic value of cone-beam computed tomography (CBCT) in patients who underwent a stapedotomy.
**Purpose/Objectives:** Over the years, radiological imaging is increasingly used for diagnosis, staging, prognosis and postoperative complications in patients with otosclerosis. CBCT is an emerging technique in the field of otolaryngology because of its reduced radiation exposure, fewer artefacts and higher resolution. This retrospective case review was performed with the aim to assess the diagnostic value of CBCT in the preoperative assessment of otosclerosis.
**Methods and materials:** A total of 89 consecutive patients (95 ears) who underwent a stapedotomy between 2017 and 2020 and were preoperatively scanned by a CBCT device because of audiological suspicion of otosclerosis, were included. The temporal bone CBCT scans were conducted on a Newtom 5G XL CBCT device with a voxel size of 0.1 mm. Axial, coronal as well as double oblique reformations were performed. Images were reviewed by one of three experienced head and neck radiologists. Findings on CBCT were categorized according to Veillon's classification of otosclerosis. Finally, intraoperative findings were correlated to the CBCT images.
**Result:** In 88 ears presence of an otosclerosis focus was confirmed intraoperatively. In this group, 87 ears (98.86%) were correctly radiologically diagnosed as otosclerosis. One case of preoperatively diagnosed tympanosclerosis was also surgically confirmed. In the remaining 6 ears, surgery demonstrated stapes ankylosis without the presence of an otosclerotic focus.
**Conclusion:** CBCT is very sensitive in the preoperative diagnosis of otosclerosis, with considerably low radiation dose. Preoperative CBCT scanning performed in all cases suspected for otosclerosis can confirm the diagnosis in 98.86% of the cases and can improve patient counseling.

### Automatic detection of inflammatory maxillary sinus lesions on low-dose cone-beam CT using convolutional neural network

*Authors:* *Lun M. Wong, Kuo-Feng Hung, Ai Qi-Yong, Yui Ya Leung, Ann Dorothy King*

**Keywords:** Maxillary sinus, mucosal thickening, mucosal retention cyst, convolutional neural network, CBCT

**Short summary:** Evaluating the health of the maxillary sinus is essential prior to planning sinus-related dental surgeries, such as sinus augmentation and apical surgery of the upper posterior teeth. Mucosal thickening (MT) and mucosal retention cysts (MRC) are common in the maxillary sinuses. The presence of MT and MRC frequently complicates the surgical procedures and may increase the risk of post-operative sinusitis. In this study, a three-stepped automatic algorithm based on convolutional neural network and support-vector regression was developed to segment and diagnose MT and MRC in the maxillary sinus from quarter-dose cone beam computer tomography. The algorithm showed an area under the curve (AUC) of 0.86 and 0.83 for the detection of MT and MRC, respectively.

**Purpose/Objectives:** Detecting mucosal thickening (MT) and mucosal retention cyst (MRC) in the maxillary sinus is an essential step in planning various sinus-related dental surgery. This study aims to propose an algorithm based on the convolutional neural network (CNN) for the automatic segmentation and diagnosis of MT and MRC in the maxillary sinus using quarter-dose CBCT images.

**Methods and materials:** Patients scanned between Feb-2016 and Oct-2020 were retrospectively reviewed, 300 cases were included. The CBCT of all patients were annotated by an expert to identify the air-space, MT and MRC in their maxillary sinuses. The patients were divided into training, testing and validation datasets at roughly 7:2:1 ratio (Table 1). The automatic algorithm comprised three steps, described in Figure 1. For each CBCT input, the air-space, MT and MRC were segmented using V-Net. From these segmentations, two indices, proportional to the probability of having MT and/or MRC were predicted for each sinus. Diagnosis was deduced using thresholds determined by the Youden index. Sensitivity, specificity and AUCs were reported. The segmentation performance was evaluated using Dice similarity score (DSC).

**Result:** The median and inter-quartile range of DSC obtained by the V-Nets in segmenting the air-space, MT and MRC were 0.98 (0.97-0.99), 0.61 (0.47-0.73) and 0.72 (0.42-0.87), respectively, in the first two steps. Using these segmentations, the support-vector regression model achieved a sensitivity, specificity and AUC of 89 %, 71 % and 0.86, respectively for detecting MT; and 86 %, 84 % and 0.83 respectively for detecting MRC on the testing dataset. The receiver operator characteristic (ROC) curve was plotted (Figure 2).

**Conclusion:** CNN can segment the maxillary sinus MT and MRC lesions, but it showed limitations in differentiating between MT and MRC. Support-vector regression using the volume, roundness and perimeter of the CNN segmentations had the potential in aiding the diagnosis of MT and MRC. In conclusion, an automatic algorithm based on CNN was proposed to detect MT and MRC on low-dose CBCT, which showed AUCs of 0.86 and 0.83 respectively.

Table 1: Patients demographics. The numbers shown were counts of sinuses. Some sinuses have both mucosal thickening (MT) and mucosal retention cysts (MRC). Kruskal-Wallis revealed no statistically significant differences.

| | Training (n=432) | Testing (n=108) | Validation (n=60) | *p*=value |
|---|---|---|---|---|
| **Healthy** | 215 | 49 | 23 | .22 |
| **MT** | 146 | 38 | 25 | .35 |
| **MRC** | 113 | 29 | 21 | .49 |

Figure 1

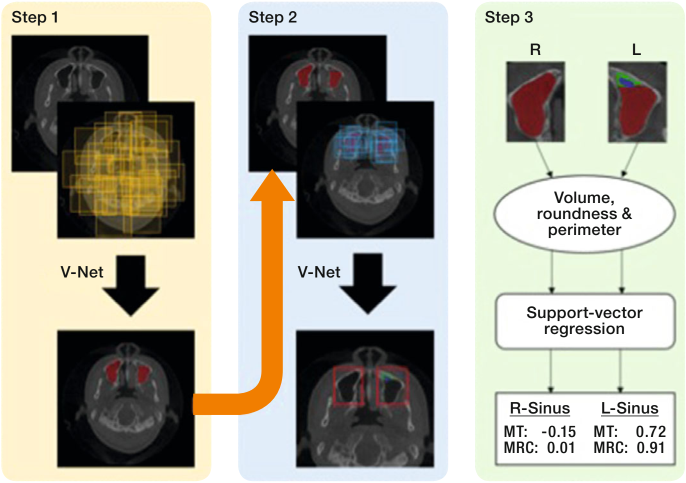

Figure 2: ROC curve for detection of MT and MRC

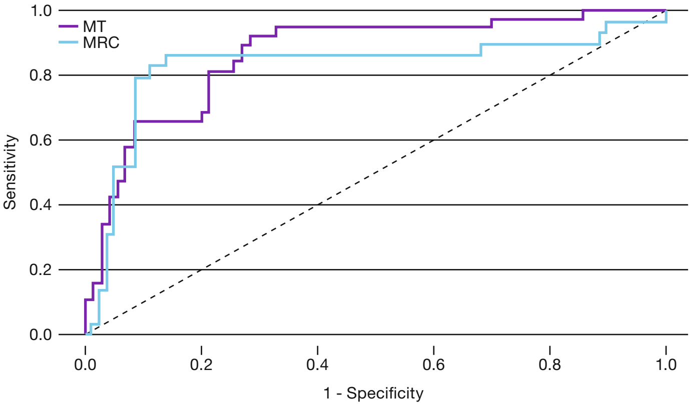

### Predicting Extranodal Extension in Metastatic Cervical Lymph Nodes of Head Neck Squamous Cell Carcinoma by Imaging Characteristics

*Authors: Yeliz Pekçevik, Tuğçe Hancıoğlu, Aslı Irmak Akdoğan, Hilal Şahin, İbrahim Çukurova, İlker Burak Arslan, Ulku Kucuk, Sumeyye Ekmekci*

**Keywords:** head and neck, squamous cell carcinoma, extranodal extension, computed tomography, magnetic resonance imaging

**Short summary:** Extranodal extension (ENE) has been associated with poor prognosis in squamous cell carcinomas of the head and neck. Identifying ENE by use of imaging modalities in the preoperative period, affects treatment strategy.

- On logistic regression analysis, perinodal fat stranding and a longest nodal diameter (> 2cm) showed statistically significant association with histopathologically proven extranodal extension (odds ratio[OR], 3.8 [p=0.002]; OR, 4.7 [p=0.017], respectively).
- Interobserver agreement for gross invasion was the highest among the investigated imaging criteria (kappa value = 0.731)
- Gross invasion showed the highest specificity for determination of ENE (96 %).

**Purpose/Objectives:** Extranodal extension (ENE), defined as the spread of tumor cells from metastatic lymph node beyond its capsule into the perinodal tissue, is a poor prognostic factor for head and neck squamous cell carcinomas (HNSCC). Identifying the ENE preoperatively, affects treatment planning and prognosis of the patients. We aim to determine the predictive imaging characteristics of the ENE and to investigate the interobserver agreement among different experience levels.

**Methods and materials:** The images of the patients, underwent neck dissection and had metastatic cervical lymph nodes of HNSCC were enrolled into the study. In correlation with pathology results, the radiologic investigations were evaluated by a single researcher for the number and levels of metastatic lymph nodes. In order to predict ENE, 106 lymph nodes of 53 patients were blindly reviewed by three radiologist that had one, two and ten years of experience in head and neck imaging. Metastatic lymph nodes evaluated for necrosis, irregularity, gross-invasion and perinodal fat stranding. The overall impression of the radiologist regarding the ENE was also noted. Sensitivity, specificity, odds ratios based on logistic regression and interobserver agreement of ENE status were calculated.

**Result:** Of 106 lymph nodes 22 (20.7 %) were positive for ENE. Smoking was associated with the presence of the ENE. Irregular contour had the highest sensitivity and gross invasion had the highest specificity for determination of ENE. The sensitivity and specificity of overall radiologists' impression were 39.3 % and 82 %, respectively. With a substantial kappa value of 0.731, interobserver agreement for gross invasion was the highest among the investigated imaging criteria. On logistic regression analysis, perinodal fat stranding and a longest nodal diameter above 2 cm were associated with the histopathologically proven ENE.

**Conclusion:** Presence of the irregular contour a metastatic lymph node had the highest sensitivity for determination of ENE. Perinodal fat stranding and a longest nodal diameter above 2 cm may be predictor for the ENE. In addition to its highest specificity in determination of the ENE, gross invasion was the best parameter with a highest interobserver agreement.

| | Pathologically proven ENE | | | |
|---|---|---|---|---|
| Variables | Sensitivity | Specifity | PPV | NPV |
| Irregular contour | 78.6 | 48.0 | 63.0 | 66.5 |
| Perinodal fat stranding | 57.1 | 74.0 | 59.4 | 72.1 |
| Gross invasion | 25.0 | 96.0 | 87.6 | 53.2 |
| Radiologists' impression | 39.3 | 82.0 | 71.0 | 54.7 |

PPV: positive predictive value; NPV: negative predictive value

| | Multivariate analysis | | |
|---|---|---|---|
| Variables | OR | 95 % CI | p value |
| Perinodal fat stranding | 3.8 | 0.116-0.601 | 0.002 |
| Largest diamater (>2 cm) | 4.7 | 0.061-0.758 | 0.017 |

Multivaraible logistic regression analysis; OR: odds ratio; CI: confidence interval

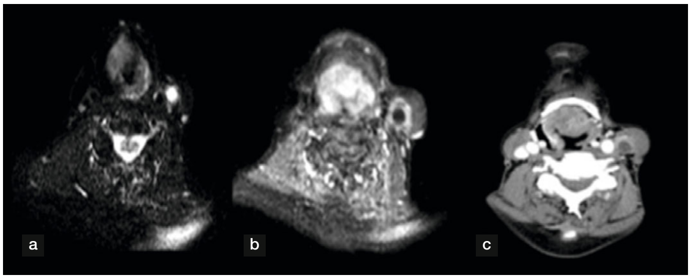

### Photoacoustic imaging of the oral cavity

*Authors: Olivia Francies, Andrew Plumb, Nam Huynh, Rehman Ansari, Paul Beard*

**Keywords:** photoacoustic, oral cavity, cancer
**Short summary:** The multiplicity of oral cavity diseases, including inflammation, dysplasia and malignancy, can be difficult to diagnose and oral squamous cell carcinoma in particular is a major health burden. High-resolution imaging would benefit the characterisation of subtle mucosal changes and detection of early malignancy, crucial for prompt treatment and improved survival. Current well-established imaging modalities e.g. ultrasound, magnetic resonance imaging, have limitations including operator-dependency and visualising submillimetre structures. Photoacoustic imaging (PAI) can non-invasively visualise the microcirculation by delivering laser pulses to tissue, which induces ultrasound wave emission. These are reconstructed into high-resolution 3D images based on the optical absorption properties of the tissue. We present in vivo images of the oral cavity demonstrating the feasibility and hence clinical utility of PAI.
**Purpose/Objectives:** The purpose of this study was to determine if PAI, as (1) a table-top platform and (2) a novel hand-held flexible endoscopic device, is capable of successfully depicting the submucosal anatomy of the oral cavity in vivo human participants.
**Methods and materials:** Ethical permission was obtained to scan healthy volunteers. The table-top PAI device consists of a 16-beam, 200 Hz 750-850-nm pulsed excitation laser with volumetric images acquired through a 15 x 15 x 15-mm volume over 8-15 s. The dual-modality endoscopic instrument integrates miniature optical systems for PAI and traditional white light videoscopy inside a 1 mm diameter, 275 nm-long stainless-steel tube. Both devices use a Fabry-Perot (FP) ultrasound sensor: a polymer spacer sandwiched between two mirrors.
**Result:** Results of this feasibility study show that the table-top PAI device is capable of producing high-resolution images of the tongue with submillimetre vessels clearly visualised (see images attached). The novel endoscopic probe was successfully tested on phantoms as well as ex vivo tissues, with vessels as small as 50 µm clearly visualised, and is due to be tested in vivo human participants shortly.
**Conclusion:** Photoacoustic imaging can achieve rapid, volumetric and high-resolution images of the oral cavity microvasculature, which could be a valuable adjunct to the current intra-oral examination of oral cavity lesions.

Figure 1

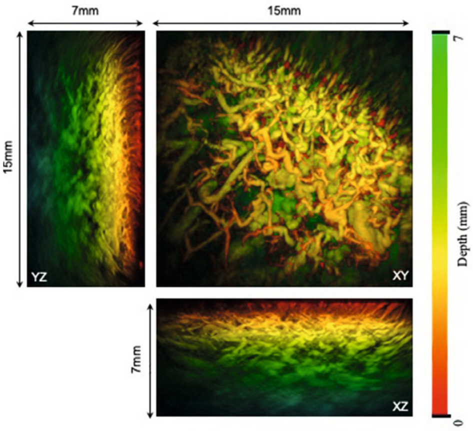

Figure 2

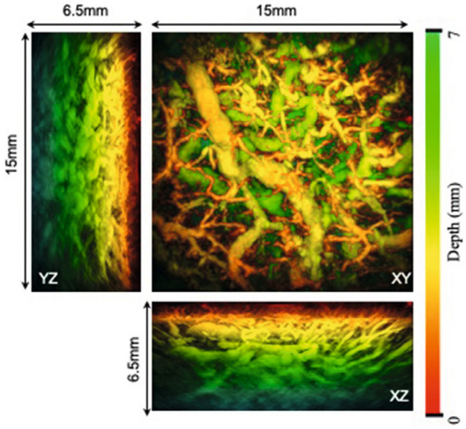

### Radiological follow-up after the bony obliteration tympanoplasty in detecting residual cholesteatoma: Towards an optimal postoperative MR imaging protocol

*Authors: Hylke van der Toom, Anja Bernaerts, Jan Casselman, Joost van Dinther, Andrzej Zarowski, Hayk Baazil, Erwin Offeciers, Bert De Foer*

**Keywords:** non-EP DW-MRI, MRI, diffusion weighted imaging, follow-up, cholesteatoma, bony obliteration tympanoplasty, BOT
**Short summary:** As there is no consensus on the most optimal follow-up protocol for non-EP DWI-MRI after cholesteatoma surgery, we retrospectively evaluated the results of this imaging modality at our institute and propose to perform an MRI at 1,3- and 5-years post-surgery.
**Purpose/Objectives:** Since its introduction in 2007, the non-EP DW MRI technique was widely adopted in the postoperative management of cholesteatoma worldwide. However, there is no consensus in literature on the most optimal follow-up imaging protocol after the canal wall-up bony obliteration tympanoplasty (CWU-BOT). On the one hand, no residual cholesteatoma should be missed but on the other unnecessary MR scans should be avoided. The aim of this study is to evaluate the postoperative results of non-EP DW MRI after CWU-BOT surgery at our institution and to propose an optimal postoperative MR imaging scheme.
**Methods and materials:** Retrospective cohort study; all 271 patients who underwent a CWU-BOT for cholesteatoma between January 2010 and January 2016 with follow-up at our institute were included.
**Result:** The median follow-up time was 60 months (inter-quartile range 56-62 months). Two hundred seventy-one patients (100%) received a 1-year MRI, 107 (39 %) a 3-year MRI and 216 (79.7 %) a 5-year MRI. Residual cholesteatoma was found in 9 cases (3.3 %), corresponding with an estimated residual rate at 5 years follow-up of 3.7 % when using Kaplan-Meier analysis. Of these 9 cases, 6 cases of residual cholesteatoma (66.7 %) were detected at the 1-year MRI (12-14 months post-surgery), 2 cases (22.2 %) at the 3-year MRI (35-39 months post-surgery) and 1 case (11.1 %) at the 5-year MRI (51 months post-surgery, in this patient no 3-year MRI was performed).
**Conclusion:** A postoperative MRI scan after 1 and 5 years is essential to detect early and late residual cholesteatoma. In our cohort, 22.2 % of residual cases were detected at the 3-year MRI. However, this percentage could potentially have been higher when all patients would have received a 3-year MRI. Therefore, in order to detect residual disease as soon as possible, we propose to perform an MRI scan at 1, 3 and 5 years after the bony obliteration tympanoplasty.

### Fully Automated 3D Vestibular Schwannoma Segmentation: A multicentre multi-vendor study

*Authors: Olaf M. Neve, Qian Tao, Stephan R. Romeijn, Yunjie Chen, Nick de Boer, Willem Grootjans, Mark C. Kruit, Boudewijn P. F. Lelieveldt, Jeroen C. Jansen, Erik F. Hensen, Marius Staring, Berit Verbist*

**Keywords:** vestibular schwannoma, segmentation, MRI, convolutional neural network

**Short summary:** For the evaluation of vestibular schwannoma (VS) progression and treatment planning, accurate measurement from MRI is important. In clinical practice, manual linear measurements are performed from MRI. Manual 3D measurement is time-consuming and 2D measurement is subjective and reflects highly variable tumour volume poorly. We developed an AI model to detect and segment VS from MRI automatically.

**Purpose/Objectives:** We present a model for the detection and segmentation of VS, based on deep learning and suited to process multi-centre, multi-vendor MR images. The model's performance is evaluated and compared to humans in an observer study.

**Methods and materials:** In total 214 cases (134 VS positive and 80 negative) with gadolinium-enhanced T1 and native T2 weighted MR images were acquired from 37 centres and 12 different MRI scanners. The intra- and extra meatal parts of the tumour were manually delineated by two observers under supervision of an experienced head and neck radiologist. Cases were divided into three non-overlapping sets (training, validation, and testing). A model was trained using 3D no-new-Unet deep learning segmentation method. In addition, an observer study was performed, in which the radiologist blinded to case information and delineation method compared model and human delineations.

**Result:** The model correctly detected VS in all positive cases and excluded the negative. Evaluation of the T1 model compared to the human delineation resulted in a Dice index 90.4±13.0, Hausdorff distance 2.12±9.32 mm, and mean surface-to-surface distance 0.49±1.52 mm. Intra and extra meatal tumour parts had Dice indices of 77.5±21.3 and 82.2±28.0, respectively. The observer study showed that in 103 out of 111 cases (93 %) the model was comparable to or better than human delineation.

**Figure 1.** Six examples of VS segmentation by our AI model and the human delineation, together with the quantitative measures.

**Conclusion:** The proposed model can accurately detect and delineate VS from MRI in a multi-centre, multi-vendor setting. As such, it is a robust tool well suited to the reality of clinical practise. The model performed comparably to human delineations in the observer study.

Figure 1

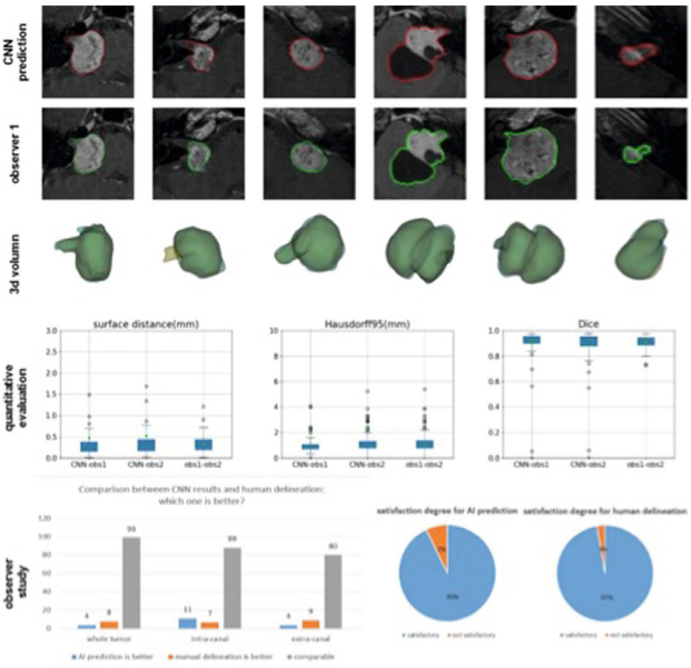

### High resolution dental MRI for endodontics – establishment of normal T1/T2 relaxation values of dental pulp and optimized MRI protocols

*Authors: Tim Hilgenfeld, Abdullah M. Saleem, Sebastian F. Schwindling, Hao Li, Johann ME. Jende, Sabine Heiland, Martin Bendszus, Alexander Juerchott*

**Keywords:** MRI, CBCT, dental, dental pulp, experimental

**Short summary:** Using novel intraoral coil designs and dedicated sequences high resolution dental MRI is capable of imaging dental pulp anatomy in a comparable quality as reference imaging modality CBCT at 3T within clinically feasible acquisition times.

**Purpose/Objectives:** To quantify T1/T2-relaxation times of the dental pulp in vivo and establish dedicated MRI protocols suitable for high spatial resolution imaging of the dental pulp.

**Methods and materials:** MR relaxometry was performed in ten healthy volunteers in 296 teeth to determine T1 and T2 relaxation times of dental pulps at 3T. Next, a human tooth phantom with an artificial pulp with comparable T1/T2 relaxation times as in vivo pulp was developed. Using that phantom signal to noise ratio (SNR), image quality and artifacts were compared for eight MRI sequences (CISS, TRUFI, DESS, FLASH, UTE, TSE, SPACE, MSVAT-SPACE) and compared to reference modality CBCT. Statistical analysis was performed using two-way ANOVA and Fisher's exact test.

**Result:** In vivo mean ± SD dental pulp T1/T2 relaxation times of 737.9±99.5 ms/171.1±35.6 ms were noted. An isotropic resolution of 0.21 mm³ and acquisition times ranging from 6:19 to 8:02 minutes were reached. Highest SNR values were achieved by UTE (mean ± SD; 99.9±4.1) followed by TSE (87.6±9.7) and CISS (83.3±9.0). Best image quality, however, revealed DESS (mean landmark score ± SD: 1.4±0.4) followed by CISS (1.5±0.3) and FLASH (1.5±0.3) with comparable results to CBCT (1.2±0.3).

**Conclusion:** Optimized MRI protocols using an intraoral coil can reveal an image quality that is comparable to reference modality CBCT at 3T within acquisition times feasible for clinical applications. Overall DESS sequence revealed best overall results, followed by CISS and FLASH sequences.

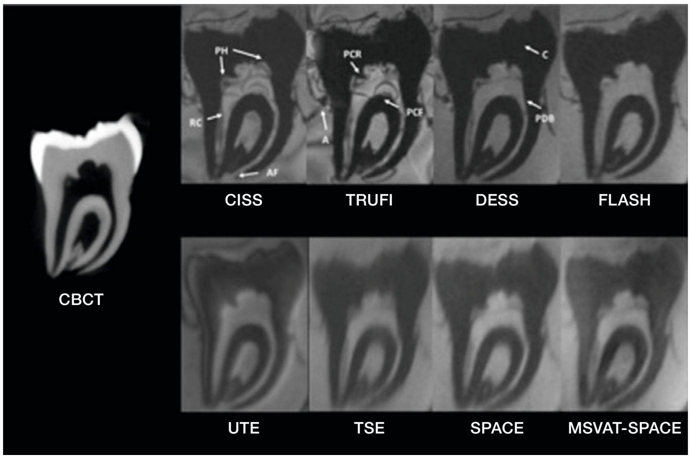

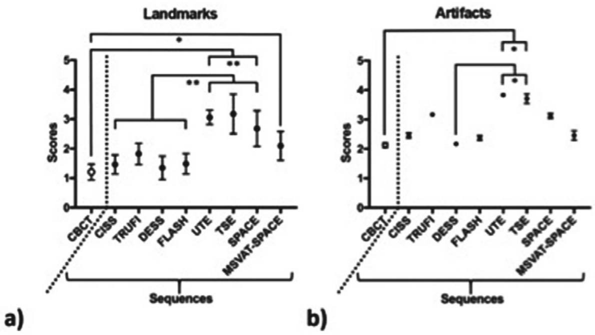

### Diagnostic value of DW-MRI and 18F-FDG PET/CT in detection of residual or recurrent tumors after radiotherapy for laryngeal and hypopharyngeal carcinoma

*Authors:* *Soung Yung Kim, Martin H. Maurer, Meinrad Beer, Christophe Schroeder*

**Keywords:** Hypopharyngeal cancer, Laryngeal cancer, Radiology, Nuclear medicine, Diagnosis

**Short summary:** Tumor persistence or recurrence following definitive radiation therapy still remains a major diagnostic and therapeutic challenge. Moreover, therapeutic options in this group of patients are limited and often not effective in oncological terms. Therefore, imaging-based evaluation plays a central role in pretherapeutic assessment. We assessed the diagnostic value of DW-MRI and 18F-FDG-PET/CT for detecting residual or recurrent tumors in patients with presenting newly developed symptoms after radiotherapy for laryngeal and hypopharyngeal carcinoma.

**Purpose/Objectives:** To compare the diagnostic accuracy of diffusion-weighted MRI (DW-MRI) and fluorine 18 fluorodeoxyglucose positron emission tomography/computed tomography (18F-FDG PET/CT) in detection of residual or recurrent tumor in patients with laryngeal and hypopharyngeal carcinoma after radiotherapy.

**Methods and materials:** 30 consecutive patients presenting with newly developed symptoms after radiotherapy for laryngeal (n=22) and hypopharyngeal (n=8) carcinoma who underwent DW-MRI and PET-CT within a period of three months were included. DW-MRI was evaluated qualitatively by visual assessment analysing high b-value images and the corresponding ADC map. PET-CT was evaluated measuring SUV related to body weight, 5 (g/ml) was taken as a cut-off to determine the presence or absence of tumor.

**Result:** Of the 30 patients, histopathology classified 20 as positive and 10 as negative for tumor. DW-MRI detected tumor in 18/20 patients and correctly excluded tumor in all negative patients. 18F-FDG PET/CT detected all tumors and was falsely positive in one case. The sensitivity and specificity of DW-MRI was 90 % and 100 %, while that of 18F-FDG PET-CT was 100 % and 90 %, respectively.

**Conclusion:** DW-MRI and 18F-FDG PET-CT had identical diagnostic accuracies in detection of residual or recurrent tumor, with DW-MRI being more specific and 18F-FDG PET-CT more sensitive in this small group of patients.

### MRI findings in pediatric neck infections – a comparison with adult patients

*Authors:* *Janne Nurminen, Jaakko Heikkinen, Tatu Happonen, Jarno Velhonoja, Heikki Irjala, Tero Soukka, Lauri Ivaska, Kimmo Mattila, Jussi Hirvonen*

**Keywords:** Magnetic resonance imaging, Emergency medicine, Neck, Infection, Pediatric

**Short summary:** The anatomic and physiologic differences between the necks of children and adults lead to different manifestations of deep neck infections. There are no prior published systematic evaluations of MRI findings of pediatric deep neck infections. Most literature, diagnostic algorithms, and recommendations are related to the use of CT as the primary modality among children when diagnostic imaging is required. We found that MRI is a feasible and accurate method for diagnosing pediatric deep neck infections. Magnetic resonance imaging (MRI) may serve as an alternative to computed tomography (CT) as the primary imaging modality.

**Purpose/Objectives:** The aim of this retrospective cohort study was to characterize the MRI findings among children with neck infections and to compare them with those found among adult patients. We further compared the MRI findings with surgical reports as a reference standard to calculate the diagnostic accuracy of MRI in the detection of neck abscesses in children and explored the prognostic values of MRI findings among these patients.

**Methods and materials:** We conducted a retrospective cohort study focusing on pediatric patients who had undergone an MRI of the neck soft tissues between 1 April 2013 and 31 December 2018.
Inclusion criteria were: 1) emergency MRI findings indicating an infection; 2) infection as the final clinical diagnosis; 3) diagnostic image quality verified by the radiologist reading the study; and 4) age under 18 years. Patient record data, including surgery reports, were compared with the MRI findings.

**Result:** A total of 45 children participated. Compared with adults, the children had a higher incidence of retropharyngeal infections and lymphadenitis, and a lower incidence of peritonsillar/parapharyngeal infections (Table 1). Examples of pediatric suppurative lymphadenitis are given in Figure 1 (patients A, B and C). MRI showed evidence of an abscess in 34 children. Of these 34 patients, 24 underwent surgery, which confirmed an abscess in 21 patients but no abscess in three patients. In addition, three patients underwent surgery without MRI evidence of abscess, and an abscess was found in one of these cases. The measures of diagnostic accuracy among the children were: sensitivity 0.97, specificity 0.77, positive predictive value 0.89, negative predictive value 0.91, and accuracy 0.89 (Table 2).

**Conclusion:** Emergency neck MRI is a feasible and diagnostically accurate imaging method for diagnosing pediatric neck infections. According to our data and the previous literature, its diagnostic accuracy is superior to that provided by CT. Additional benefits of MRI, especially among children, include the lack of ionizing radiation. Thus, MRI should be considered as the primary imaging modality in children when imaging is needed in the diagnostic process of deep neck infections. In younger age groups, retropharyngeal space infections and suppurative lymphadenitis are emphasized, reflecting the anatomical differences between children and adults. These differences require special attention and radiological interpretation skills.

Table 1: Characteristics of pediatric neck infection patients (N=45).

| | |
|---|---|
| **Age (median, range)** | 9.0 |
| **Female N (%) / male N (%)** | 14 (31) / 31 (69) |
| **Duration of symptoms at admission (mean, days)** | 4.8 |
| **Fever (body temp. over 37.5 °C) prior to imaging [N (%)]** | 18 (40) |
| **WBC (mean, x 109/L)** | 14.2 |
| **CRP value at admission (mean, mg/L)** | 78.8 |
| **Hospital stay (mean, days)** | 3.9 |
| **Required intensive care [N (%)]** | 10 (22) |
| **Duration of intravenous antimicrobial medication (mean, days)** | 4.1 |
| **Primary infection location [N (%)]** | 45 (100) |
| retropharyngeal / mediastinal | 11 (24) |
| infected lymph node / cyst | 11 (24) |
| peritonsillar / parapharyngeal | 10 (22) |
| sublingual / submandibular | 7 (16) |
| superficial | 3 (7) |
| parotid | 2 (4) |
| masticator / buccal | 1 (2) |
| **Maximum abscess diameter (mean, mm)** | 31 |

Table 2: Diagnostic accuracy of MRI in neck abscesses in pediatric and adult patients using surgery report as reference standard.

| | TP | FN | FP | TN | Se | Sp | PPV | NPV | FPR | FNR | Acc |
|---|---|---|---|---|---|---|---|---|---|---|---|
| **PEDIATRIC N=38** | 24 | 1 | 3 | 10 | 0.96 | 0.77 | 0.89 | 0.91 | 0.23 | 0.04 | 0.89 |
| **ADULT N=290** | 191 | 4 | 11 | 84 | 0.98 | 0.88 | 0.95 | 0.95 | 0.12 | 0.02 | 0.95 |

TP=true-positive; FN=false-negative; FP=false-positive; TN=true-negative; Se=sensitivity; Sp=specificity; PPV=positive predictive value; NPV=negative predictive value; FPR=false-positive rate; FNR=false-negative rate; Acc=accuracy

Figure 1

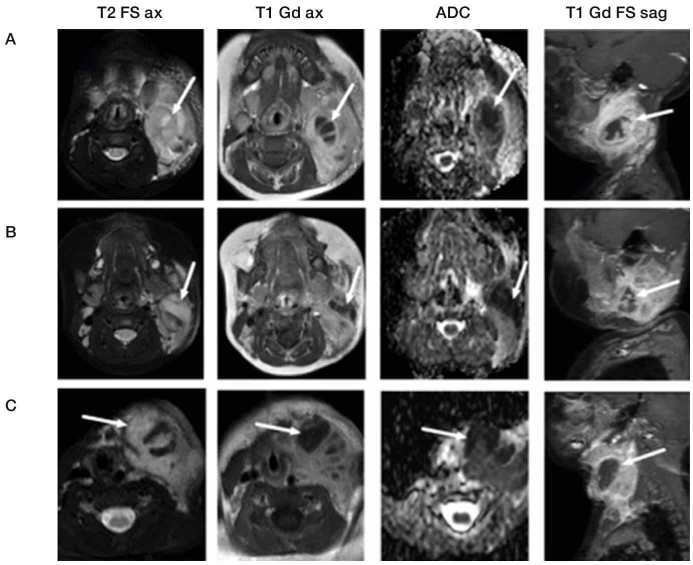

### Endovascular Approach in Impending or Acute Carotid Blowout Syndrome in Head and Neck Malignancies

*Authors:* *Sedat Kandemirli, Omer Fatih Nas, Baris Korkmaz, Mehmet Fatih Inecikli, Muhammed Fırat Oztepe, Cem Bilgin, Bahattin Hakyemez*

**Keywords:** carotid blow out, embolization, endovascular treatment
**Short summary:** Carotid blowout syndrome (CBS) can be a life-threatening event in patients with head and neck malignancy with an incidence rate varying between 2.9-10 %. Surgical exploration and repair can be challenging due to necrotic nature of the tissues, especially in cases with prior surgery and irradiation. Endovascular approach can be used in management of CBS.
**Purpose/Objectives:** To present our endovascular treatment experience in carotid blowout syndrome (CBS) in patients with head and neck malignancy.
**Methods and materials:** A retrospective review of patients that underwent endovascular treatment for carotid blowout syndrome between 2012 and 2019 in our interventional radiology department identified a total of 17 patients. We retrospectively reviewed the imaging data and clinical findings. Coiling, detachable balloons or covered stents were used for endovascular treatment. We reviewed rate of infarction, infection and rebleeding during follow-up. The distribution of cancer was as follows: larynx in 9, nasopharynx in 4, oral cavity in 3 and esthesioneuroblastoma in 1. Fourteen patients had received radiotherapy. 9 patients had impending CBS and 8 patients had acute CBS. On DSA, vascular abnormality was localized to ECA in 5, CCA in 5, ICA in 4, costocervical trunk in 1 and brachiocephalic trunk in 1.
**Result:** Vascular abnormality included aneurysm formation in 9 case, pseudoaneurysm in 5 case, laceration in 1 case and cutaneous fistula in 1 case. Endovascular treatment was performed with aneurysm coiling in 5 case, parent vessel occlusion (PVO) in 10 case and covered stent deployment in 1 case. In the esthesioneuroblastoma case, no vascular abnormality was identified on DSA and prophylactic PVO of sphenopalatine artery was performed. The treatment was technically successful in 14 cases, whereas additional parent vessel occlusion or stent placement were required in the remaining 3 cases. During the post-procedural period, 3 cases had infection and 4 patients suffered from cranial infarcts. Rebleeding episodes were encountered in 8 cases after a mean duration of 35 days.
**Conclusion:** Endovascular treatment in the form of covered stent, aneurysm coiling or PVO for impending or acute CBS can achieve high technical success rates with moderately increased complication rates. There is relatively increased rate of rebleeding episode in the short-term follow-up.

### Scientific Posters

### Our experience of ultrasound-guided fine-needle aspiration sample adequacy during training lists

*Authors:* Joshua Frost, Joshua Lauder

**Keywords:** Fine-needle aspiration, Adequacy, Cytology, Ultrasound

**Short summary:** Many serious and malignant disorders can initially present as a head or neck mass. Patients presenting with a head or neck mass may require a tissue biopsy to make a histological diagnosis and treatment plan. Fine-needle aspiration (FNA) cytology is a valuable technique in the work-up of nodules and masses arising within the head and neck. It is widely accepted as a safe, accurate, sensitive and cost-effective diagnostic procedure with relatively low risk of complications. However, diagnostic yield varies with experience, technique (type of needle, needle gauge, path chosen), tissue and type of lesion. This study therefore aims to assess the adequacy of head and neck FNA samples taken by consultants and speciality registrars for cytological diagnosis.

**Purpose/Objectives:** The aim of this study was to determine the success rate of ultrasound guided FNA of head and neck masses, assessing the percentage of specimens which were adequate and representative for cytological diagnosis. The objective of this study was to ensure that the adequacy of FNA samples of head and neck masses meets national standards. In the UK, the Royal College of Radiologists (RCR) recommends 70% of all FNAs should be diagnostic on cytology assessment.

**Methods and materials:** A picture archiving and communication system (PACS) was used to collate a list of 50 patients from January 2021 to March 2021 that had undergone FNA of a head and neck mass. The patients' ages ranged from 14 - 86 years. The hospital's radiology reporting system was accessed to determine which physician performed the FNA, the site of FNA, the number of passes, the needle gauge used and use of local anaesthetic. The hospital's electronic pathology reporting system was then accessed to determine the adequacy of 53 FNA samples for histological diagnosis.

**Result:** 53 samples were recorded from 50 patients. The origins of the tissue were thyroid (30), lymph node (12), salivary gland (10) and base of tongue (1). Overall, 79.2 % (42/53) of the samples were classified as adequate for cytological diagnosis. The adequacy rate for each tissue type was 76.7 % (23/30) for thyroid tissue, 75 % (9/12) from lymph node tissue and 90 % (9/10) from salivary gland tissue. FNA samples were taken by a mix of radiology registrars and five consultant radiologists. Registrars together performed almost half of FNAs (26), with an adequacy rate of 73.1 %, whereas collectively, consultant radiologists' samples (27) had an adequacy rate of 85.2 %.

**Conclusion:** Current practice is in keeping with RCR guidance, though there is a clear disparity between the adequacy rate of consultants versus the adequacy rate of speciality registrars. This is understandable given the level of experience, though changes can be implemented to improve these rates. Suggested changes include increasing the number of passes registrars perform to ensure they gather an adequate sample. Furthermore, if the sample is particularly difficult to retrieve, a smaller gauge needle may be used to optimise the capillary effect.

### MRI for detecting cholesteatoma of the middle ear: The importance of observer´s reliance level in different DWI-sequences

*Authors:* Benjamin Henninger, Christian Kremser, Volker Schartinger, Herbert Riechelmann, Joachim Schmutzhard, Natalie Fischer

**Keywords:** MRI, cholesteatoma, DWI

**Short summary:** Diffusion-weighted imaging (DWI) in Magnetic resonance imaging (MRI) has developed as an important tool for the detection of cholesteatoma. Various DWI sequences are available and have shown promising results. In our study, EPI RESOLVE DWI and coronal non-EPI HASTE DWI were both reliable techniques for diagnosing primary and recurrent cholesteatoma. The reliance score is an important predictor for reliability of MRI in detection of cholesteatoma and could be an interesting tool for surgeons decision on indicating a second-look operation.

**Purpose/Objectives:** This study aims to evaluate the importance of the observer´s reliance level for the detection of cholesteatoma using different DWI sequences.

**Methods and materials:** Forty patients (14 female and 26 male; age range 16-87 years; mean age 44 years) were included into the study meeting the following criteria: 1) chronic otitis media, 2) preoperative MRI including various DWI sequences, 3) middle-ear surgery. Surgical validation confirmed the cholesteatoma diagnosis. The MRI protocol contained the following sequences: (1) axial and (2) coronal echo-planar imaging (EPI) readout-segmented (RESOLVE) DWI with TRACE acquisition, (3) coronal non-EPI half-Fourier acquired single-shot turbo spin-echo (HASTE) DWI. Cholesteatoma diagnosis was based on standard diagnostic criteria for cholesteatoma with DWI. Additionally the radiologists were asked to grade personal reliance to their diagnosis using the Likert-like scale; from 1 (=very insecure) to 5 (=very secure). The radiologic diagnosis was correlated to the surgical result.

**Result:** Axial and coronal RESOLVE DWI showed a sensitivity of 77.3 % and a specificity of 72.2 %, respectively. The mean reliance was 3.9 for axial and 3.8 for coronal images. HASTE DWI had a sensitivity/specificity of 81.8 %/66.7 % with the highest reliance of all evaluated sequences (4.4). Cases with a reliance level of 5 showed a sensitivity / specificity of 100 % in all sequences. A reliance level of 5 was given in the axial and coronal RESOLVE DWI in 32.5 % of cases, and in the HASTE DWI in 57.5 %.

**Conclusion:** The evaluated DWI sequences showed comparable results for detecting middle ear cholesteatoma. The reliance level significantly improved the predictor of cholesteatoma disease with MRI technique.

### CT-based skeletal muscle mass estimation in head and neck cancer (HNC)

*Authors:* *Stephanie Mangesius, Daniel Dejaco, Lukas Haider, Julian Mangesius, Hanna Kranebitter, Marcel Kloppenburg, Matthias Santer, Elke R. Gizewski, Herbert Riechelmann, Gerlig Widmann*

**Keywords:** HNC, skeletal muscle mass, planimetry, volumetry, survival
**Short summary:** In head and neck cancer (HNC), skeletal muscle mass (SMM) before and after treatment influences overall survival. The reference method to radiologically assess the mass of skeletal musculature in HNC represents time-consuming volumetry of the sternocleidomastoid muscle (SCM) from CTs.
**Purpose/Objectives:** Whether the volume of SCM can be predicted from simpler and manually measurable planimetry has not been explored yet.
**Methods and materials:** A sample of 100 patients with advanced HNC previously treated with primary radiochemotherapy was randomly drawn from our institutional cancer registry. Manual volumetry from pre- and post-treatment diagnostic CTs of SCMs were performed with commercially available software (Mint Lesion, Germany) at the level of C3. SCM's planimetric measurements including long- and short axis were further derived. Unadjusted, linear regression models were used to explore the correlation of volumetric with planimetric measurements. Planimetric measurements in $mm^3$, confidence intervals (CI) and R2values were provided.
**Result:** Of 100 patients with advanced HNC, mean age was 70 + 10 years; 26 were female. All planimetric measurements significantly correlated with volumetric measurements (all p<0.001). For pre-treatment CTs, short- and long-axis planimetry for the right SCM were 0.48 $mm^3$ (CI 0.31-0.65; R2=0.25) and 0.32 $mm^3$ (0.26-0.37; R2=0.56), for the left SCM 0.44 $mm^3$ (0.31-0.57; R2=0.31) and 0.29 $mm^3$ (0.24-0.35; R2=0.54), for post-treatment CTs 0.39 $mm^3$ (0.20-0.59; R2=0.14) and 0.31 $mm^3$ (0.25-0.36; R2=0.58) and 0.61 $mm^3$ (0.43-0.79; R2=0.32) and 0.25 $mm^3$ (0.20-0.30; R2=0.48), respectively.
**Conclusion:** Long-axis planimetry of SCM at the level of C3 appeared to significantly correlate with SCM's volume with acceptable model fit ($R^2 \geq 0.5$). Simpler planimetry may aid in exploring the clinical relevance of skeletal muscle mass as prognosticator for HNC-patients.

### Late labyrinthine enhancement – a useful tool in differentiating inner ear diseases

*Authors:* *Anja Giesemann, Kerstin Willenborg, Athanasia Warnecke, Friedrich Götz, Desiree Grosser, Paulo Dellani, Heinrich Lanfermann, Thomas Lenarz*

**Keywords:** temporal bone, labyrinth, MRI, Enhancement
**Short summary:** Late labyrinthine enhancement as a visualisation of Blood labyrinth barrier impairement might be useful in some cases of unexplained hearing loss. We encountered it in 13 cases of patients scanned for suspected Menière disease and the corresponding diagnoses turned out to be vestibular neuritis, Menière disease, otosclerosis and strongly suspected autoimmune disease. However half of the cases remained unexplained and the hearing loss was suggested to be due to inflammatory or infectious origin. The Enhancement was not remarkable in early post contrast images and best seen on late 3D Real IR images.
**Purpose/Objectives:** To describe the imaging findings in cases of late enhancement of the labyrinth as found in patients evaluated for endolymphatic hydrops using MRI.
**Methods and materials:** A total of 13 patients out of 200 consecutively scanned patients for endolymphatic hydrops were found to have late labyrinthine enhancement (11 female, 2 male, average age 52,1 years) Intervention(s): Hydrops MRI was performed in all patients. Main Outcome Measure(s): pattern and degree of late enhancement, Signal intensity ratio (SIR)
**Result:** Bilateral n=6 and unilateral n=7 late labyrinthine enhancement was found. Complete enhancement of cochlea and vestibule was found in four patients (two bilateral, two unilateral cases). The rest of the patients presented with partial enhancement of either the cochlea or the vestibule or only parts of the cochlea. SIR demonstrated a marked difference in enhanced areas of the cochlea especially in late Flair images. Thus enhancement was easier detectable and in many cases only detected on these enhanced Flair images. The diagnosis was verified in two cases of vestibular neuritis, 3 cases with Ménière's disease and in one case of otosclerosis. In another case, autoimmune related inner ear disease was strongly suspected based on clinical data.
**Conclusion:** Late enhancement might detect BLB impairment more reliably than early post contrast images. Although the diagnostic procedure is quite time consuming it should be advocated specifically for cases with suspected inflammation or unclear cause of inner ear related symptoms.

### Educational Posters

### A pictorial review of the helpfulness of diffusion weighted imaging sequences in head and neck pathology

*Authors: Nirav Kaneria, Hannah Marsh*

**Keywords:** Diffusion weighted imaging, Head and neck, Helpfulness
**Short summary:** Given the compact nature of the anatomical structures in the head and neck, incidental pathology is difficult to identify in certain situations unless being specifically looked for. We present cases where reviewing the diffusion weighting imaging (DWI) has helped identify pathology which is pertinent and of relevance to the patient.
**Learning objectives:**
1. Describe the generally accepted uses for diffusion imaging in the head and neck.
2. Demonstrate cases where DWI has identified clinically relevant pathology, not easily identifiable on alternative MRI sequences.

**Background:** Head and neck pathology can be subtle given the compact anatomical structure of the neck. When not being specifically searched for and without distinguishing T1 and T2 signal characteristics, some pathology can be missed. The DWI and Apparent diffusion coefficient (ADC) sequences are known to have uses, for example in primary tumour and cholesteatoma assessment, but its use in other areas is not widely described in the literature.
**Imaging findings and procedure details:** The generally accepted uses of diffusion imaging will be demonstrated. In addition to this and for the primary aim of the poster we present cases highlighting pathology that could only have been incidentally picked up due to diligently reviewing the DWI sequence. The DWI acts as a beacon to a specific site which can then be scrutinised on alternative sequences. The cases being described highlight how the reporting radiologist can find pathology which is important and incidental.
**Conclusion:** The DWI sequence is helpful in localising incidental unexpected pathology which can then be scrutinised on the alternative sequences to obtain a differential diagnosis.

### Extramedullary Plasmacytoma of the Thyroid Cartilage: A Case Report

*Authors: Yensa Rodríguez, Maria Pané Foix*

**Keywords:** MRI, cholesteatoma, DWI
**Short summary:** Thyroid Cartilage, Extramedullary Plasmacytoma, Laringeal tumor
**Short summary:**
- Plasma cell myeloma is the most common plasma cell neoplasm, it is characterized by the presence of multiple lesions in the bone marrow. A single isolated lesion may occur either in bone (solitary plasmacytoma of bone) or in soft tissue (extramedullary plasmacytoma -EMP).
- Most cases of EMP occur in the supraglottic region but plasma cell neoplasm that involves the thyroid cartilage is extremely rare.
- Men are twice as often affected by EMP than women,whereas the $6^{th}$ decade of life is the most probable time for disease occurrence.
- Imaging techniques (CT or MRI), are helpful in order to clarify the local involvement of anatomical structures as well as to exclude further osseous and soft-tissue lesions and lymphadenopathy.

**Learning objectives**
- To present radiological a histopatological imaging finding of Extramedullary Plasmacytoma of the Thyroid Cartilage.
- To be familiar with a rare tumor of the head and neck region, clinical behavior and therapeutic management.
- This case could be instructive for the differential diagnosis of laryngeal lesions due to the rarity of these neoplasms and the unusual localization of the extramedullary plasmacytoma tumor.

**Background:** A 63 year-old man presented with complaint of increasis hoarseness and foreign body sensation on the airway for 7 months. Direct laryngoscopy showed mobile vocal cords with diffuse submucosal swelling. Right false vocal cord was hypertrophic without airway obstruction. A contrast enhanced computed tomographic (CT) scan revealed an enhanced expansile mass originating within the thyroid cartilage predominantly in the right side; on MRI the mass showed restriction diffusion. Diagnosis was confirmed fine-needle aspiration (FNA) biopsy was performed of the laryngeal mass. The patient was treated with radiation therapy (total of 40 Gy in fractionated doses) with slight resolution of the mass.
**Imaging findings and procedure details:**
**Figure 1:** CT: Hyperdense mass contained within arcs of expanded and eroded thyroid cartilage (yellow arrows) with homogeneous enhancement resulting in a slight glottic stenosis. Some parts of the cartilage is still bordered by a thin calcified layer. MR: Bulging mass originating from the thyroid cartilage shows high signal on T1 WI and homogeneous enhancement (orange arrows) with restricted diffusión (green arrows).
**Figure 2:** Microscopic pictures. H&E stain shows sheets of large atypical lymphoid cells with plasmacytic differentiation with abundant cytoplasm and large nuclei. ICH with positive staining for MUM1 and CD 138. Kappa light chains show a significant difference in staining intensity between kappa and lambda chains in favor of lambda chains demonstrating that the tumor is lambda-restricted.
**Conclusion:** 18 % of the Extramedullary plasmacytoma (EMP) occur in the head and neck. Thyroid cartilage is a rare site of EMP and evaluation for underlying multiple myeloma (MM) must be made in all patients. In patients with primary EMP, radiotherapy is therapy of choice. In patients with secondary EMP, treatment is directed towards the underlying MM and therapy of the EMP is palliative. The imaging findings of extramedullary plasmacytoma in the larynx are non-specific. The major role of imaging is to confirm the presence of a tumor mass and show the extent of the lesion.

### MRI studies in parotid tumors: Differentiation between benign and malignant lesions. What the radiology trainee should know

*Authors: Soung Yung Kim, Martin H. Maurer, Christophe Schroeder, Meinrad Beer*

**Keywords:** Magnetic resonance imaging, parotid gland tumor, differential diagnosis

**Short summary:** MRI is the imaging modality of choice for evaluation of suspected parotid gland lesions and differentiation between benign and malignant lesions. This educational poster provides a comprehensive overview of imaging features of most common parotid lesions. Radiology residents and subspecialized fellows should know the characteristic imaging features and be familiar with other differential diagnoses.

**Learning objectives:** To comprehend the imaging features for differentiation between benign and malignant lesions in parotid grands.

**Background:** Parotid tumors are complex, involving a wide variety of benign and malignant lesions. Clinical assessments have limitations in diagnosing malignant parotid neoplasms. In addition, fine needle aspiration biopsy is sometimes inconclusive and may provide insufficient results. Therefore, diagnostic imaging, especially MRI has assumed a major role for assessing the location and malignancy of the tumor.

The purpose of this educational poster is to provides a brief overview of imaging features of most common parotid lesions and to evaluate which MR imaging findings are best able to predict malignant histology. Furthermore, our poster aimed to describe the possible differential diagnosis of various parotid tumors.

**Imaging findings and procedure details:**

1. The role of MR imaging in assessment of parotid tumors
2. Benign salivary gland tumors
3. Imaging features of benign parotid tumors A. Pleomorphic adenoma, B. Cystadenolymphoma: Warthin´s tumor, C. Oncocytoma, D. Infantile Hemangioma, E. Lipoma, F. Benign lymphoepithelial lesion
4. Malignant salivary gland tumors
5. Imaging features of malignant parotid tumors A. Mucoepidermoid carcinoma, B. Adenoid cystic carcinoma, C. Acinic cell carcinoma, D. Carcinoma-ex-pleomorphic adenoma, E. Lymphoma, F. Metastasis
6. Localization of parotid tumors in relation to the intraparotid facial Nerve
7. Local extension and invasion
8. Peri neural spread
9. Detect nodal metastases
10. Differential diagnosis of parotid tumors

**Conclusion:** Most benign and malignant parotid tumors can be discriminated by their appearance on MR imaging. Radiology residents and fellows should know the characteristic imaging features and be familiar with the possible differential diagnoses.

Figure 1

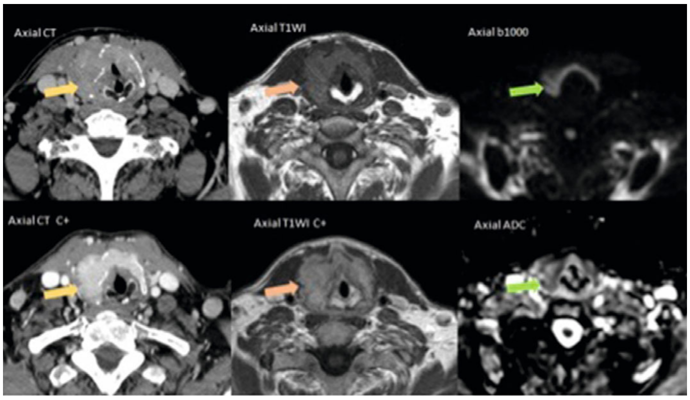

Figure 2

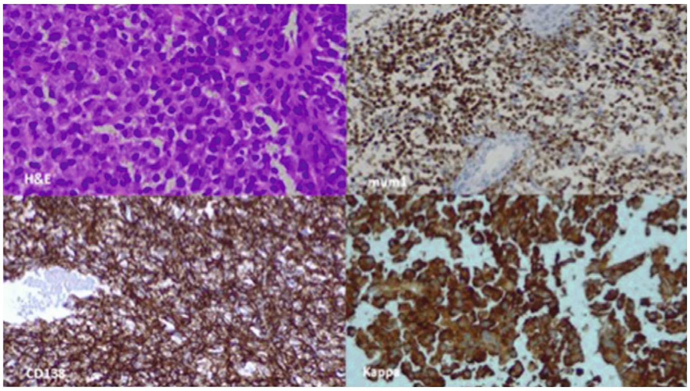

### Detecting perineural spread: A journey along V and VII cranial nerves

*Authors:* *Hunor Sükösd, Davide Maraghelli, Cosimo Nardi*

**Keywords:** perineural tumor spread, trigeminal nerve, facial nerve, perineural invasion, adenoid cystic carcinoma, squamous cell carcinoma, fossa pterygopalatina
**Short summary:** This education poster presentation offers a systematic approach to the anatomy and efficient detection of perineural spread along the most common pathways involved in tumors of the head and neck. It also includes the zonal classification and therapeutic consequences of such finding.
**Learning objectives:** Given the importance of perineural tumor spread in disease staging and therapeutic planning in head and neck tumors, detection of this pathology is crucial even in the setting of non-specialist centers. This presentation serves as a survival guide and should improve understanding and detection of this disease.
**Background:** Perineural spread is a tumoral invasion along a nerve sheath away from the primary site, most often involving the branches of V and VII nerves in case of head and neck tumors. The incidence in this region varies ranging from 14 % to 63.2 %, most commonly presenting with cutaneous malignancies like melanoma, mucosal primaries such as squamous cell carcinoma and salivary gland malignancies, most of all adenoid cystic carcinoma. Other malignancies such as adenocarcinoma, myeloma, lymphoma, and leukemia can present perineural tumor spread as well, although less frequently. Detecting perineural involvement requires extensive knowledge of anatomy and specialized radiology skill set. A systematical approach may provide a better and easier understanding of the frequent patterns and signs of this pathology.
**Imaging findings and procedure details:** Schematics and radiologic images are provided supporting better understanding and recognition of the anatomy and the tumoral spread along nerves. Magnetic Resonance Imaging is considered the most sensitive method for its detection, although in many cases indirect signs of invasion can also be identified on CT images. The most significant signs are the thickening and enhancement of the nerves, the enlargement of the nervous foramen and the obliteration of the perineural fat planes.
**Conclusion:** Perineural tumor spread is a complex disease requiring an extensive specialized knowledge of anatomy and radiology. Although the detection of this pathology greatly defines therapeutic planning, diagnostics of these patients are not always possible in highly specialized centers. Improving basic knowledge and understanding of patterns and signs through a systematic approach may greatly improve detection and therapeutic results as well.

### Silicone lymphadenopathy mimicking malignant cervical lymphadenopathy

*Authors:* *Edward Lightfoot, Brook Adams, Victoria Birkett*

**Keywords:** Cervical lymphadenopathy, axillary lymphadenopathy, supraclavicular lymphadenopathy, silicone lymphadenopathy, ultrasound, MRI, Histology, Misdiagnosis
**Short summary:** Silicone lymphadenopathy is a rare but recognised complication of procedures utilizing silicone, and has been most commonly associated with rupture of Poly-Implant Prosthesis (PIP) breast implants. We present a case of a 33 year old woman initially referred for investigation of neck swelling, with MRI confirming large volume cervical and axillary lymphadenopathy. Subsequent ultrasound and histological investigations confirmed silicone lymphadenopathy related to a ruptured PIP implant.
**Learning objectives:**
- Recognise silicone lymphadenopathy as a rare but important non-neoplastic cause of cervical lymphadenopathy.
- Review the classical imaging and histological findings of silicone lymphadenopathy

**Background:** Silicone breast implants are one of the most commonly performed cosmetic procedures. Complications are rare but include local inflammatory reactions, connective tissue disease, auto-immune disease and malignancy, and complications are more common with PIP implants. PIP implants were withdrawn from the UK in 2010 after it was found they had been fraudulently manufactured with unapproved silicone gel and were far more prone to rupture than other breast implants.
**Imaging findings and procedure details:** Imaging findings related to implant rupture and silicone leak are well known to breast radiologists, but are likely less well recognised by their general or head & neck colleagues. MRI imaging demonstrates large volume non-specific cervical and axillary lymphadenopathy. Subsequent ultrasound assessment demonstrates the classic snowstorm appearance within the enlarged lymph nodes.
**Conclusion:** Silicone lymphadenopathy is a rare but recognised cause of cervical lymphadenopathy. Bauer et al in 2011 demonstrated that 6/18 women with confirmed silicone lymphadenopathy presented with supraclavicular nodes and suspicion of implant rupture was raised in only 39 % of cases, and median time from implant rupture to lymph node biopsy and implant removal was 107 months. This entity is therefore an important non-neoplastic cause of neck lymphadenopathy that head and neck radiologists should be aware of, as initial misdiagnosis can cause unnecessary anxiety and inappropriate investigations.

### Catastrophic Bleeding from the Middle Ear Cavity after ear grommet insertion: A Case Report

*Authors:* *Talaat Youssef, Moegamad Ashraf Ederies*

**Keywords:** Jugular Bulb, Ear Gormment, CT, Anatomic Variant
**Short summary:** A 5-years old child was admitted to our hospital for elective procedure of adenotonsillectomy and ear grommet insertion. Upon insertion of right ear grommet, massive bleeding came from right ear
**Learning objectives:** Describe how anatomic variants of temporal bone such as bony dehiscent of high riding jugular bulb can cause massive bleeding.
**Background:** We report a case of massive bleeding from right middle ear following grommet insertion.
**Imaging findings and procedure details:**
**Procedure Details:** Upon insertion of right ear grommet, massive bleeding came from right ear estimated around 100 ml. This was treated immediately by compressive packing was applied and secured with crept band. Patient remained stable, kept intubated. However, the patient was massively re-bleeded after removing compressive packs. CT scan was performed initially to diagnosis bleeding source. A follow-up CT scan was performed 2-3 weeks later.
**Imaging Findings:** CT scan of the temporal bone demonstrated deeply inserted grommet extending to anomalous variant of high riding and dehiscent right jugular bulb. Ear packed with surgical fibrillar then floseal hemostatic matrix applied. Follow-up CT showed no evidence of acute intracranial haemorrhage or venous sinus thrombosis.
**Conclusion:** Surgeons and radiologists should be aware of anatomic variants of temporal bone such as dehiscent and high riding jugular bulb that may result in catastrophic events and massive bleeding

### Find your way through the labyrinth – learning temporal bone anatomy from pathologies

*Authors:* Vanessa Meihui Khoo, Jocelyn Yen Ling Wong, Betsy Kar Hoon

**Keywords:** Middle ear, inner ear, anatomy, high resolution imaging, pathology
**Short summary:** Advancements in CT and MRI have enabled detailed evaluation of the anatomical structures and detection of pathologies involving the temporal bone. This poster will facilitate learning of normal anatomy of the middle and inner ear structures with side-by-side comparison against abnormal findings through a series of cases.
**Learning objectives:**
- To familiarize readers with key anatomical structures within the middle and inner ear on high resolution MRI and CT scans, with the use of annotations. Provide various imaging examples of pathology to reinforce anatomical knowledge.
- To compare and contrast the normal from the abnormal imaging findings.

**Background:** In recent years, the use of dedicated high-resolution magnetic resonance imaging (MRI) protocols has enabled better detection of congenital and acquired abnormalities in the middle and inner ear. The anatomy of the middle and inner ear can be challenging to learn but a good working knowledge of these structures becomes more essential than before. We aim to make this learning process easier using annotations to highlight key structures and showcase important pathologies affecting them.
**Imaging findings and procedure details:** High resolution CT and MR images demonstrating normal anatomy of middle and inner ear structures and their pathology.
**Conclusion:** Comprehensive knowledge of the middle and inner ear anatomy is a prerequisite for detection of radiological findings or pathologies. The annotation of the relevant anatomical structures affected by the disease will make this an interactive learning. By showcasing both normal and abnormal images, readers will be able to compare and contrast and gain greater insight.

### Common patterns of perineural tumour spread in head and neck malignancy: A pictorial review

*Authors:* Michael Couzins, Sobana Battison, Madan Ethunandan

**Keywords:** perineural spread, carcinoma, perineural
**Short summary:** We present a pictorial review of the common patterns of perineural tumour spread in head and neck malignancy
**Learning objectives:** To understand the common nerves affected by perineural spread in head and neck malignancy and the imaging features that demonstrate this disease process.
**Background:** Perineural tumour spread is a well-known mechanism of tumour extension in head and neck malignancy. With up to 40% of cases clinically asymptomatic, imaging plays a vital role in its diagnosis and can be easily missed if not carefully evaluated for. The presence of perineural tumour spread reduces prognosis, increases the risk of recurrence and limits management options.
**Imaging findings and procedure details:** The features of perineural tumour spread include abnormal nodularity, thickening and enhancement of the nerve, enlargement of the nerve canal or foramina and secondary effects such as muscular denervation. Furthermore, although on a pathological level perineural tumour spread is continuous, it can appear discontinuous on imaging, therefore good anatomical knowledge and thorough assessment along the entire course of the cranial nerves is essential.
**Conclusion:** In this pictorial review, we present imaging findings of common nerves affected by perineural tumour spread within the head and neck.

### Role of Multidisciplinary team meetings (MDT) in accurate staging of complex head and neck cancers including junctional tumours

*Authors:* Swapndeep Singh Atwal, Imroz singh Sachdev, Neeru Tomer

**Keywords:** junctional tumours, multidisciplinary team meeting, staging
**Short summary:** Accurate staging of head and neck cancers is vital for appropriate treatment planning and fair assessment of prognosis. Whilst imaging plays an important role is predicting staging sometimes it is challenging to reach accurate staging with imaging alone and multidisiplinary inputs play a major role in such scenarios.
**Learning objectives:** Understanding challenges in staging of complex head and neck cancers like junctional cancers. Evaluating the role of MDT discussion in reaching correct staging in complex oncology cases.
**Background:** Imaging plays a vital role in evaluation of head and neck cancers to delinate complete extent of the disease and thefore predicting staging of the cancer which guides management and estimating prognosis. However, there are certain scenarios where staging is challenging based on imaging alone and clinical findings correlation is very essential to reach correct staging. Multidisciplinary team meetings provide such platform to discuss imaging, clinical observations and histological findings and correlating multidisplinary findings to estimate correct disease burden which enables optimal treatment planning.
**Imaging findings and procedure details:** The presentation demonstrates the importance of MDT discussions in reaching the appropriate staging in complex head and neck cancer. This is illustrated by multiple real scenarios where MDT discussions and addition of clinical observations made a significant difference in reaching optimum staging of cancers.
**Conclusion:** MDT discussions are very important in accurate staging of head and neck cancers. Correlation of clinical findings along with radiological features are essential in reaching correct diagnosis, more so in complex oncology scenarios such as junctional tumours.

### Imaging in pulsatile tinnitus: A practical approach, checklist and pictorial review

*Authors:* Shikha Sivasankara Pillai, Mohamed Nasr

**Keywords:** Pulsatile tinnitus, CTA/ CTV, MRI/MRA
**Short summary:** Pulsatile tinnitus is a perception of rhythmic sounds in the ear, frequently occurring in synchrony with vascular pulsations. This poster discusses the role of imaging in the evaluation of pulsatile tinnitus and describes the causes as a pictorial review.
**Learning objectives:**
1. To describe an imaging algorithm in pulsatile tinnitus with emphasis on the role of CTA/CTV.
2. To provide a pictorial review of common and interesting differentials.
3. To propose an imaging checklist and a search pattern that helps to avoid common pitfalls in diagnosis.

**Background:** Pulsatile tinnitus is a common clinical entity with a profound psychosocial impact on the quality of life of the affected person. The possible differentials range from “touch me not” vascular variants to treatable causes like dural arteriovenous fistulas, carotid dissection, glomus tumors and meningiomas. The goal of imaging is to detect potentially treatable causes. Recent NICE guidelines recommend contrast enhanced CT imaging of the temporal bone for evaluation of suspected osseous or middle ear abnormalities. If the clinical/audiological assessments are normal, MRA/ MRI of head, neck, temporal bone and IAM are recommended. In our institution, CT angiogram/ venogram is the first line of imaging with MRI /MRA reserved for problem solving
**Imaging findings and procedure details:** We retrospectively reviewed the CT and MR images performed for pulsatile tinnitus in our institution over the last three years (2019-2021) and present a pictorial review of common as well as uncommon differentials.
**Conclusion:**
1. The goal of imaging is to find a treatable cause of pulsatile tinnitus and the radiologist must be aware of the key findings and the potential pitfalls in making the diagnosis,
2. CTA/ CTV is a quick, cost effective and easily available examination which can evaluate the arterial, venous, middle and inner ear structures in a single sitting. However MR may be used complementarily to solve diagnostic dilemma in negative cases.
3. Systematic review of images using a search pattern and an imaging checklist help to improve the diagnostic yield.

### Cochlear Hypoplasia in Syndromic Hearing Loss: An Imaging Review

*Authors: Luiz Ricardo Araújo Uchôa, Hugo Luis Vasconcelos Chambi Tames, Marcelo Delboni Lemos, Bruno Casola Olivetti, Maíra Oliveira Sarpi, Carlos Toyama, Regina Lucia Elia Gomes, Eloísa Maria Mello Santiago Gebrim*

**Keywords:** cochlear hypoplasia, syndromic hearing loss, head and neck radiology

**Short summary:** A wide spectrum of radiological abnormalities can be found in the cochlea of subjects with syndromic causes of hearing loss. Different and sometimes specific types of cochlear hypoplasia (CH) have been described as characteristic of some rare syndromes, including the Branchio-Oto-Renal (BOR) spectrum, Waardenburg and Walker-Walburg Syndromes.

**Learning objectives:** To review syndromic causes of sensorineural hearing loss that present cochlear hypoplasia and to demonstrate in detail the image findings of the CH that are specific in rare syndromes, with the purpose of broadening among radiologists and enhance the current understanding of distinct imaging features in patients with rare syndromic hearing loss and CH.

**Background:** CH can be defined as a cochlea of abnormally small external dimensions and according to the latest classification system, there are four different groups of CH. Several rare syndromes with sensorineural hearing loss have an overlap of clinical characteristics and some present with cochlear hypoplasia typical with imaging features that may assist in the diagnosis as Branchio-Oto-Renal spectrum, Waardenburg and Walker-Walburg.

**Imaging findings and procedure details:** BOR Syndrome: described as tapered basal turn and hypoplastic middle and apical turns with an anterior offset, this deformity gives an "unwound" appearance to the cochlea which is very sensitive and specific for the diagnosis. Waardenburg Syndrome: cochlea was often hypoplastic, fits the definition of CH type 3, specifically moderate to severe hypoplasia and hypoplastic modiolus. Walker-Warburg Syndrome: have a highly characteristic CH type 4, characterized by a normal basal turn and extremely hypoplastic upper turns that appeared offset anteriorly with respect to the basal turn.

**Conclusion:** A complete knowledge of the image findings in the CH is critical for the optimal assessment and diagnosis management of the patients with rare syndromic hearing loss.

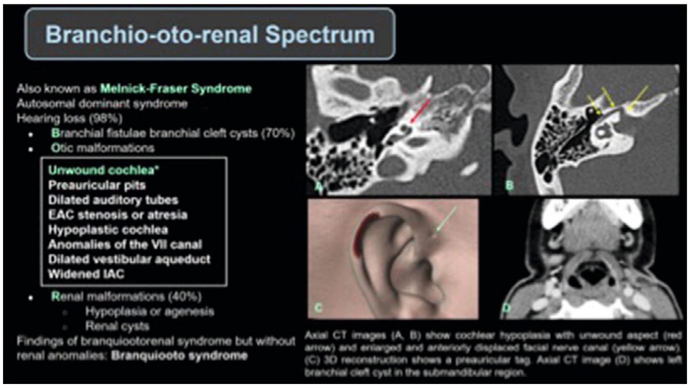

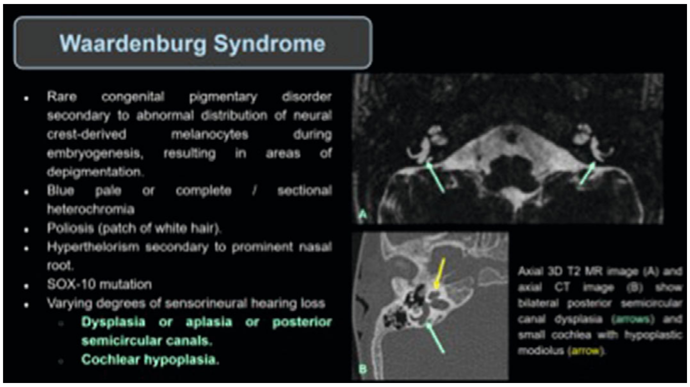

### Unexpected orbitofrontal occurrence of a classic cranial base case

*Authors: Mirjam Wielema, Auke P. A. Appelman*

**Keywords:** Cholesterol granuloma, Orbital pathology, Orbitofrontal lesion

**Short summary:** This poster presents an example of a classical radiological appearance of a cholesterol granuloma at an uncommon location. This T1 and T2 hyperintense non-enhancing lesion usually presents at the petrous apex. However, it can arise in the orbito-frontal region and should therefore be in the differential diagnosis of extra-conal masses. Pathology is very specific and surgical resection is curative.

**Learning objectives:** Cholesterol granuloma classically appears at the petrous apex. Multiple cases of orbito-frontally located cholesterol granulomas have been described and this entity should therefore be in the differential diagnosis of an orbital mass. MRI classically shows a both T2 and T1 hyperintense sharply demarcated non-enhancing mass, without diffusion restriction and with bony remodeling on CT.

**Background:** Cholesterol granuloma is a lesion with an uncertain etiology. It is thought to be the result of an inflammatory reaction to blood breakdown products and usually occurs at the petrous apex. The middle ear cavity is another possible location. However, in the literature there are multiple cases described of an orbito-frontal occurrence, especially at the lacrimal fossa. A systematic review described 172 orbitofrontal cases (1934-2015) [Hughes 2016]. Seven recent articles describe another 25 cases [Yan 2015/Shirao 2015/Imre 2015/Ugga 2017/Alkatan 2018/Rong 2019/Yokoi 2020/Guradarrama-Ortiz 2021]. We present a case from our practice involving a 50 year old male who presented with left upper eyelid swelling, ptosis and double vision of the left eye. There was no relevant medical history or previous trauma.

**Imaging findings and procedure details:** MRI showed an extra-conal sharply demarcated lobulated T1 and T2 hyperintense mass with remarkable T1 and T2 hypo-intense rim in the superolateral quadrant, measuring 28x24x21 mm (Fig.1). The lesion was non-enhancing, non fat suppressed (Fig.2) and showed facilitated diffusion on DWI. There was no connection to the lacrimal gland or extraocular muscles (Fig.2). No intracranial dural enhancement or invasion was present. CT showed osseous remodeling with a focal defect at the frontal sinus and anterior skull base (Fig.3). Given the unusual location for a cholesterol granuloma, at first instance dermoid cyst and mucocele were considered in the differential diagnosis. Surgical (curative) resection showed a classic cholesterol granuloma consisting of old hematoma, cholesterol crystals with giant cells, fibrosis and ferrous pigmentation.

**Conclusion:** Known classic locations of the cholesterol granuloma are the petrous apex and middle ear cavity, however multiple cases of cholesterol granuloma in the orbito-frontal region have been reported. Given their classical MRI characteristics, orbitofrontal cholesterol granuloma should be easy to recognize, once you are aware that they can appear in this region.

Figure 1

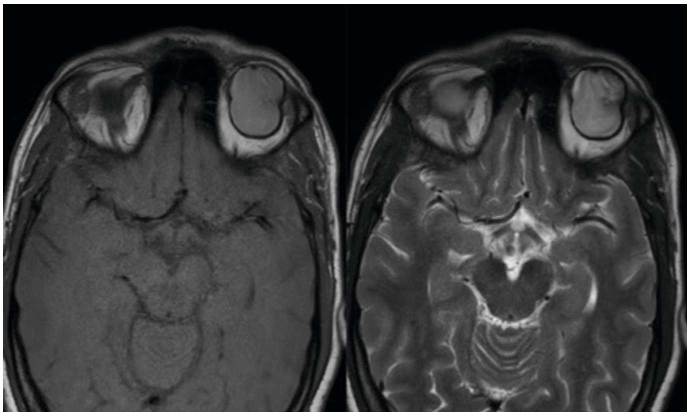

Figure 2

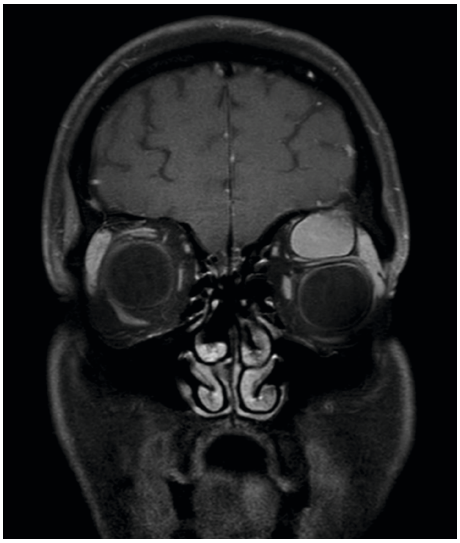

Figure 2

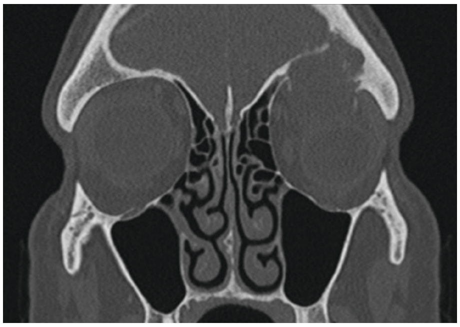

### US-Guided Percutaneous Microwave Ablation for Benign Thyroid Nodules – Make it Easy!

*Authors: Maria Leonor da Silva Dias Fernandes Pita, André Peixoto, Sara Peixoto, João Leitão*

**Keywords:** Microwave Ablation, Thyroid, Minimally Invasive, US-Guided, Benign Nodule, Radiofrequency, Ethanol, Thermal Percutaneous Procedures

**Short summary:** Benign thyroid nodules are extremely common. Some can cause pressure symptoms, be cosmetic annoying or be related to autonomously functioning problems. Although Surgery and Radioiodine therapy are the gold standard treatment in such instances, modern non-surgical minimally invasive percutaneous ultrasound-guided options are becoming appealing alternatives as for Microwave Thermal Ablation (MWA). It does not need hospitalization, has lesser risks like recurrent laryngeal nerve palsy, usually causes only minimal scars, and does not result in iatrogenic hypothyroidism. The Authors describe the basics of MWA, compare it to other procedures that are available such as radiofrequency ablation (RFA), percutaneous laser ablation (LA), high-intensity focused ultrasound (HIFU) and ethanol ablation in cases of cystic lesions (EA).

**Learning objectives:** What are the Principles and Technical Background on Microwave Ablation? How about Patient's Selection Criteria? Evidence Based Medicine on MWA – What do we already know? What is still there to acknowledge? Can we compare MWA with other Percutaneous Ablation Techniques?

**Background:** Benign thyroid nodules are extremely common in the general adult population ultrasound examinations, and many of them are asymptomatic and really do not need any treatment. Nevertheless, some can cause pressure symptoms, be cosmetic annoying or be related to autonomously functioning problems. In those instances, Surgery and Radioiodine are the gold standard treatment nowadays, but modern non-surgical minimally invasive options are being more and more an appealing alternative. Microwave is one of the thermal ablation procedures available options, as Radiofrequency, Laser and High-intensity Focused Ultrasound therapy.

**Imaging findings and procedure details:** High-quality evidence on MWA is still limited and we are looking forward for the next years publications to gain more confidence. In the Authors Institution, it was recently started (2021), and that was the inspiration to this Educational Poster. We purpose to resume the state-of-the-art on the basics Imaging findings and procedure details of MWA in the universe of thyroid thermal Ablation Techniques, illustrated with some of our cases that are being analyzed in a prospective way, and plan to have more results to show in a near future.

**Conclusion:** There is an increasing interest in Microwave Ablation and is becoming gradually an important tool to treat several benign thyroid nodules. However, longer-term outcomes require further validation and is still ATA recommended as a second-line modality in most instances. This work tries to make it technically as simple as possible in order to fortunately gather more and more Radiologists interested in it, being able to offer MWA to their selected patients that really can benefit from it in the near future years to come.

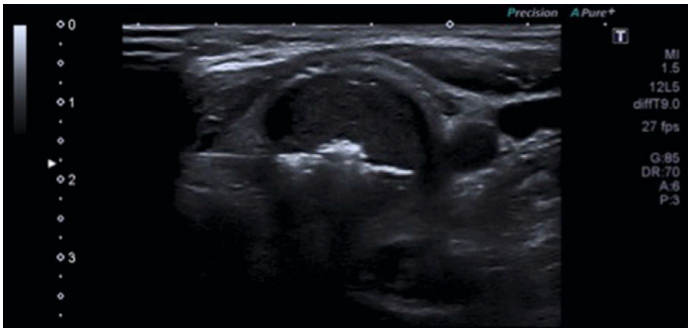

### Radiological features of endolymphatic sac tumours: A tertiary centre experience along with a brief review of literature

*Authors:* Anne Péporté, Thomas Hundberger, Christian Rothermundt, Claudia Hader

**Keywords:** ELST, VHL, MRI

**Short summary:** Endolymphatic sac tumours (ELST) are rare, locally aggressive tumours of the temporal bone. They are seen in young and middle-aged adults. ELST may occur sporadically, however are often associated with Von Hippel-Lindau (VHL) disease (seen in almost 10% of cases). Hence, it is beneficial to routinely screen patients with VHL for this tumour. Patients may present with hearing loss, tinnitus, vertigo, and facial paresis. ELST is classically located in the retrolabyrinthine temporal bone, involving the endolymphatic sac. These are hypervascular tumours, often showing destructive bony changes as well as reactive new bone formation. Diagnosis is established on clinical, radiological, and pathological correlation. This educational review describes our tertiary institutional data on ELST along with a brief review of imaging literature.

**Learning objectives:**
- To review the clinico-pathological features of ELST -To highlight the radiological features of ELST on CT and MRI
- To familiarize trainee radiologists with certain helpful diagnostic pearls and potential pitfalls/imaging mimics

**Background:** Using the search words 'ELST' we came across 5 cases diagnosed with ELST on imaging in our institutional PACS from the years 2011-2021. All of these were associated with VHL and all were detected on MRI. Additional HRCT was performed in 2 of 5 patients. The age range was 24–54 years (M:F – 3:2). Hearing deficit was the most common clinical presentation. All ELST were primarily diagnosed by imaging and confirmed by histopathology after resection. All patients underwent surgical resection. All patients are on continuous long-term follow-up scans not only to detect recurrence of ELST, but also to detect VHL-associated brain parenchymal hemangioblastomas.

**Imaging findings and procedure details:** Our routine MR screening protocol for VHL/ELST consists of T2W TSE sagittal and axial images and axial diffusions-weighted images of the entire brain, axial T1W VIBE images and post-contrast fat-saturated T1W VIBE images of the temporal bones. HRCT of the temporal bones is performed using axial and coronal reconstructed images before and after contrast administration with a slice thickness of 0.6 mm. On CT, ELST shows heterogenous attenuation typically with destructive changes in the adjacent petrous bone. Reactive new bone formation is noted. The jugular foramen is generally spared, a fact that helps to distinguish ELST from glomus jugulare tumours. On MRI, ELST often show T1 hyperintensity due to haemorrhagic changes. Intense post-contrast enhancement is seen due to high vascularity.

**Conclusion:** ELST are rare tumours of the temporal bone. Despite their rarity they should be part of the differential diagnosis of tumours arising from the petrous temporal bone and occupying the cerebellopontine angle, especially if associated with destructive bony changes. Radiologists should maintain a high degree of suspicion especially in cases of VHL. Familiarity with their clinico-radiological features would help both the clinician and radiologist to arrive at an accurate diagnosis and optimize treatment with a multidisciplinary approach.

### A case of a facial pseudomass: Diagnosis, imaging and management in unilateral temporalis muscle hypertrophy

*Authors:* Mervyn Chong, David Annan, Ashok Adams, Tamer Theodossy

**Keywords:** Temporalis hypertrophy, facial pseudolesion, masticator muscle pathology

**Short summary:** Unilateral hypertrophy of the temporalis muscle is an uncommon condition that may present as a facial pseudomass. This can pose a diagnostic challenge as the differential diagnosis includes both benign and malignant entities. Radiological imaging is frequently utilised in the diagnostic work-up of a facial mass and a biopsy may be required to exclude a sinister diagnosis. Management is aimed at symptomatic relief and achieving a satisfactory cosmesis for the patient. We present an educational poster on this condition using imaging from a clinical case.

**Learning objectives:**
- To appreciate current concepts in the aetiology of unilateral temporalis muscle hypertrophy.
- To review the MRI appearances of unilateral temporalis hypertrophy and the differential diagnoses for a unilateral facial mass in this region.
- To understand the approach to clinical and imaging diagnosis of unilateral temporalis hypertrophy and the available management options.

**Background:** Unilateral masticator muscle hypertrophy is a relatively uncommon condition and there are very few case reports of unilateral hypertrophy of the temporalis in the head and neck literature. The aetiology of acquired masticator muscle hypertrophy remains controversial although parafunctional jaw activity such as bruxism and temporomandibular joint disease are frequently considered to be implicated. It is advised that in patients presenting with apparent unilateral enlargement of the temporalis that aggressive conditions such as sarcoma, leukaemia or lymphoma, and inflammatory myopathies are also considered in the differential diagnosis.

**Imaging findings and procedure details:** Imaging may be utilised in the diagnostic work-up, most commonly with ultrasound and/or MRI. In cases where there is diagnostic uncertainty or concern for a sinister process then a biopsy may be performed. The treatment options for temporalis muscle hypertrophy include symptomatic relief with oral analgesia, muscle reduction surgery and local injection of botulinum toxin.

**Conclusion:** In our educational exhibit we will discuss the condition using a case investigated with MRI in which surgical biopsy was subsequently performed to confirm the diagnosis. We will also discuss the differential for abnormalities involving and relating to the temporalis with examples including lymphoma.

### Know your Luc's from Ludwig's? Learn the various routes of infection spread within the head and neck.

*Authors:* Dominic Fong Kuan Ting, Jocelyn Yen Ling Wong, Betsy Kar Hoon

**Keywords:** Head, neck, compartment, anatomy, infection, abscess, routes of spread

**Short summary:** A wide range of infections can occur within spaces of the head and neck. An understanding of the common routes for the spread of infection is vital to delineate the extent of infection, as well as for evaluation of potential complications. The infection can extend cranially into the intracranial space or caudally into the mediastinum. We seek to outline the anatomy and routes of infection using illustrations and annotations via a series of cases to enhance learning.

**Learning objectives:**
- To facilitate learning of the head and neck spaces, outliningfascial planes and vital structures using illustration and annotations of key anatomical landmarks.
- To familiarize the readers with potential routes for dissemination of head and neck infection upon detection of the primary source.

**Background:** The head and neck contain vital structures including the major vessels and airway which may be compromised secondary to severe head and neck infection. Infection can spread across spaces of the neck or extend superiorly or inferiorly into the intracranial space or mediastinum respectively. Good knowledge of head and neck anatomy will aid in detection of the source of infection as well as recognize its spread. This, in turn will assist clinicians in planning their surgical approach for drainage and help to anticipate potential complications.

**Imaging findings and procedure details:** High resolution CT and MR images demonstrating anatomy of various neck compartments and potential routes for dissemination of head and neck infections.

**Conclusion:** There are multiple areas where infection can occur in the head and neck. An understanding of the anatomical compartments in the head and neck and the potential routes for dissemination of infection is essential. These are depicted through a case series with illustrations and annotations in this poster, making this an interactive learning experience.

### Anaplastic thyroid carcinoma: Approach considerations and assessment of surgical resectability

*Authors:* Mariana Horta, Tiago Lopes Nunes da Silva

**Keywords:** Thyroid, oncologic, Anaplastic thyroid carcinoma

**Short summary:** Anaplastic thyroid carcinoma represents 1-2 % of all thyroid cancers and it is associated with the highest mortality risk of any type of thyroid tumour. A rapid and efficient multidisciplinary team assessment of these tumours is required, where Radiology has a pivotal role. We describe the role of imaging in the approach of anaplastic thyroid cancer and in the assessment of surgical resectability.

**Learning objectives:** To describe the role of imaging in the work up of anaplastic thyroid cancer, focusing not only in the initial diagnosis with ultrasound fine-needle aspiration and/or core-biopsy, but also in computed tomography characteristics in the assessment of surgical resectability of anaplastic thyroid carcinoma.

**Background:** Anaplastic thyroid carcinoma represents 1-2 % of all thyroid cancers and it is associated with the highest mortality risk of any type of thyroid tumour. Historically, with conventional therapies the median survival of patients following the diagnosis was of 5-6 months and patients had a 1-year overall survival of 20 %. Recent molecular target therapies such as the combination of drabafenib/trametinib showed incredible efficacy in anaplastic thyroid carcinomas that activate B-Raf Kinase (BRAF) V600 mutation (between 20-50 %). Therefore, a rapid and efficient multidisciplinary team assessment of these tumours is required, where Radiology has a pivotal role.

**Imaging findings and procedure details:** We will provide US and CT images of anaplastic thyroid carcinoma focusing in the initial diagnosis and in the assessment of the feasibility of surgical resection.

**Conclusion:** Anaplastic thyroid carcinoma is a rare and extremely aggressive thyroid tumour. Recent molecular target therapies have shown incredible efficacy in anaplastic thyroid carcinomas that activate B-Raf Kinase (BRAF) V600 mutation, changing the prognosis of these types of tumours. Therefore, a rapid and efficient multidisciplinary team assessment of these tumours is required, where Radiology has a pivotal role

### Good to hear: What surgeons want to know in pre-operative imaging of Cochlear Implants

*Authors:* Catarina Mendes Pinto, Eduarda Maria Marinho Pinto, Cristina Ramos, Bruno Moreira

**Keywords:** CT, MRI, Cochlear Implant

**Short summary:** Through this educational presentation we aim to review the pre-operative imaging approach of Cochlear Implants, namely the normal anatomy and the anatomic conditions which may have an impact on the surgical procedure.

**Learning objectives:**
- To recognize the normal anatomy of the external, middle, and inner ear on CT and MR, with a focus on potential findings that preclude the surgery.
- To understand the indications for a cochlear implant.
- To review the image characteristics of the main pathology with surgical indication.
- To identify conditions with potential impact on the surgical procedure.

**Background:** Cochlear implants are increasingly used as a powerful tool to treat severe sensorineural hearing loss, both in children and adults. Although the number of surgeries had increased in the last decade, the indications for cochlear implants remain strict and should be followed with care to ensure good post-surgical results. One of the most important steps on patient selection is the imaging workup with complementary CT and MR, once anatomic abnormalities can alter surgical planning in up to 20% of cases. Also, some absolute contraindications preclude the implantation, and radiologists have an important role to identify these situations in pre-surgical studies.

**Imaging findings and procedure details:** We present illustrative cases from our practice to review the imaging findings in the pre-operative workflow of Cochlear Implants. Normal CT and MR anatomy will be discussed with several annotated images to correctly identify middle and inner ear structures, as well as anatomic variants of the temporal bone that may influence the surgical approach. Complete labyrinthine aplasia, cochlear aplasia, and complete cochlear ossification are some of the contraindications that will be reviewed in this presentation. Also, cochlear aperture abnormalities and oval and round windows hypoplasia will be addressed. Position of the facial nerve, distance from the facial nerve and stapedic muscle, and the main measures used in the assessment of the round window aperture and visibility will also be reviewed.

**Conclusion:** Pre-operative imaging in Cochlear Implants, both with CT and MR, are crucial for surgical success. Radiologists should be able to recognize anatomical variants that may influence the surgical approach and to identify contraindications that preclude the implantation, enhancing the results and prognosis after the surgical intervention.
